# The Genome Sequence of *Polymorphum gilvum* SL003B-26A1^T^ Reveals Its Genetic Basis for Crude Oil Degradation and Adaptation to the Saline Soil

**DOI:** 10.1371/journal.pone.0031261

**Published:** 2012-02-16

**Authors:** Yong Nie, Yue-Qin Tang, Yan Li, Chang-Qiao Chi, Man Cai, Xiao-Lei Wu

**Affiliations:** Department of Energy and Resources Engineering, College of Engineering, Peking University, Beijing; Cinvestav, Mexico

## Abstract

*Polymorphum gilvum* SL003B-26A1^T^ is the type strain of a novel species in the recently published novel genus *Polymorphum* isolated from saline soil contaminated with crude oil. It is capable of using crude oil as the sole carbon and energy source and can adapt to saline soil at a temperature of 45°C. The *Polymorphum gilvum* genome provides a genetic basis for understanding how the strain could degrade crude oil and adapt to a saline environment. Genome analysis revealed the versatility of the strain for emulsifying crude oil, metabolizing aromatic compounds (a characteristic specific to the *Polymorphum gilvum* genome in comparison with other known genomes of oil-degrading bacteria), as well as possibly metabolizing *n*-alkanes through the LadA pathway. In addition, COG analysis revealed *Polymorphum gilvum* SL003B-26A1^T^ has significantly higher abundances of the proteins responsible for cell motility, lipid transport and metabolism, and secondary metabolite biosynthesis, transport and catabolism than the average levels found in all other genomes sequenced thus far, but lower abundances of the proteins responsible for carbohydrate transport and metabolism, defense mechanisms, and translation than the average levels. These traits support the adaptability of *Polymorphum gilvum* to a crude oil-contaminated saline environment. The *Polymorphum gilvum* genome could serve as a platform for further study of oil-degrading microorganisms for bioremediation and microbial-enhanced oil recovery in harsh saline environments.

## Introduction

Bioremediation has proved to be an effective method for cleaning petroleum polluted environments and has engendered intensive interest and research world-wide [1]. However, its application is still limited. One of the reasons is that the crude oil constitutes, including numerous long chain alkanes, aromatic compounds, and asphaltene, are too complex, biologically refractory and even toxic to microbes. Another reason is the possible high salinity of oil polluted environments, such as marine and coastal environments. For example, oil fields in China often locate in saline regions, and oil production wastewater is often characterized by a wide range of salinities [2]–[4]. For successful bioremediation and microbial-enhanced oil recovery [5], bacteria should therefore have the abilities both to degrade oil components and to adapt to the harsh saline environment.

Isolation of bacteria able to degrade crude oil components has been ongoing for a long time and hundreds of bacterial strains have been isolated from diverse environments including oil production water, oil-polluted soil, and marine sediment [6]–[10]. Meanwhile, several enzymes and pathways responsible for oil degradation have been found, including the integral-membrane non-heme diiron monooxygenase (AlkB) [11]–[14] and the cytochrome P450 CYP153 family-related [15], [16] metabolic pathways for degradation of medium chain length *n*-alkanes (C8–C16). Although less researches were made on enzymes that can degrade long chain alkanes (>C18), LadA and a novel AlkB-rubredoxin fusion protein coding gene have recently been identified and their ability to degrade long chain alkanes has been proved [17], [18]. Genes involved in anaerobic alkane degradation and metabolic pathways have also been identified [19]–[21].

Adaptation to the environment is important for microorganisms to survive. which is controlled by various mechanisms at genetic s level. The two-component systems (TCS), including the EnvZ/OmpR system for osmolarity sensing [22], CheA/CheY for chemotaxis [23], and DesR/DesK for thermosensing [24], for example, are found in almost all bacteria [25]–[27] and can sense and respond to environmental changes. Upon sensing, succeeding regulations are induced in the bacteria, followed by initiation of the cells to express metabolism, transportation, and other mechanisms for adapting to the environmental stresses, i.e. the accumulation of K^+^ and compatible solutes synthesis in osmotic shock [28]–[30] or the regulation of chaperone proteins to help with correct protein folding or degradation of unfolded proteins in heat shock [31].

Progress in complete genome sequencing is offering more and more information on how bacteria conduct crude oil degradation and environmental adaptation at genetic levels. For example, the complete genome sequences of *Alcanivorax borkumensis* SK2 and *Geobacillus thermodenitrificans* NG80-2 revealed their abilities to degrade a wide range of hydrocarbons and crude oil [32]. The genome sequence of the marine bacterium *Desulfatibacillum alkenivorans* AK-01 provides a blueprint for anaerobic alkane biodegradation [33]. In addition, the genome of NG80-2 is well equipped with genes encoding various transporters for efficient nutrient uptake and detoxification as well as genes for environment sensing, responses and the successive regulation of metabolism [32] that make living in oily environment easier. Similarly, the genomes of strains SK2 and AK-01 also harbor genes for responses to stresses and adaptation to marine environments. Although three complete genomes of three different oil-degrading bacteria are now known, research on genetic basis for oil degradation and environmental adaptation is just in its infancy, as the entire hydrocarbon metabolic pathway, the regulatory network, and the mechanisms of adaptation to the environment are yet to be elucidated.

Here we report the complete genome sequence of an oil-degrading bacterium, *Polymorphum gilvum* SL003B-26A1^T^, the type strain of a novel species in the recently published novel genus *Polymorphum* isolated from crude oil-contaminated saline soil in Shengli Oilfield, China [34]. It is Gram negative, facultatively anaerobic, and motile. It can grow at temperatures between 4 and 50°C, in the pH range 5.0–9.0, and at NaCl contents of 0–6% (w/v), with optimum growth occurring at 37°C, pH 6.0, and 1% (w/v) NaCl. It can grow on and produce acids from various carbon sources, and is resistant to a broad spectrum of antibiotics [34]. In addition, the strain can degrade crude oil components and utilize crude oil as its carbon and energy sources, making it a potential candidate for bioremediation and oil recovery. The genome analysis of the strain gave insights into the mechanisms of hydrocarbon degradation and metabolism and oil niche-specific stress sensing, responses, regulation, and environment adaptation.

## Results and Discussion

### General genome features of *Polymorphum gilvum* SL003B-26A1^T^

#### COG analysis

**Figure 1:**
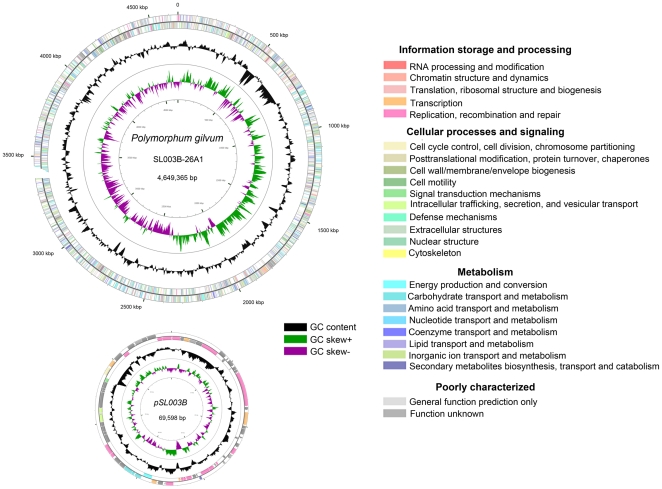
Circular chromosome of *Polymorphum gilvum* SL003B-26A1^T^. The scale on the outside indicates the size. Position 1 of the chromosome was assigned to the first nucleotide of the *dna*A gene. Rings 1 and 2 (from the outside in) indicate the genes in forward and reverse strands respectively, the colors of the genes indicate the COG categories, as indicated in the figure. Rings 3 and 4 indicate the G+C content and GC skew [(C−G)/(C+G)], respectively. Circular genome map was generated by CGview [78].

The complete genome of *Polymorphum gilvum* SL003B-26A1^T^ consists of a circular 4,649,365-bp chromosome and a 69,598-bp plasmid with G+C contents of 67.22% and 61.55%, respectively (Figure 1) [35]. The chromosome contains 4,322 predicted protein coding genes (CDS) with an average size of 955 bp, giving a coding intensity of 88.70%. Fifty tRNA genes and 2 rRNA operons were identified in the chromosome. The plasmid contains 71 predicted CDS with an average size of 869 bp, giving a coding intensity of 88.63%. Of the entire 4,393 CDS, 3,578 could be assigned to cluster of orthologous groups (COGs) (Table 1), which were analyzed to understand how *Polymorphum gilvum* SL003B-26A1^T^ deploys its genes in the genome. In addition, the 3,578 CDS could be assigned to 21 different categories (Figure 1), including those for amino acid transport and metabolism (category E, 10.35%), transcription (K, 6.88%), energy production and conversion (C, 6.45%), inorganic ion transport and metabolism (P, 5.56%), and signal transduction mechanisms (T, 5.00%).

**Table 1:** Key features of the *Polymorphum gilvum* SL003B-26A1^T^ genome.

Feature	Chromosome	Plasmid
Size (bp)	4,649,365	69,598
G+C content (%)	67.22	61.55
CDS (protein coding genes)	4322	71
Coding density	88.7	88.63
Avg of CDS length (bp)	955	869
genes with COGs	3532	46
No. of tRNA genes	50	0
No. of rRNA operons	2	0

A one-sample *t* test was used to evaluate if there were statistically significant differences of the gene abundances of each COG categories between *Polymorphum gilvum* SL003B-26A1^T^ and other genomes in the IMG genome database. The results showed that the abundances of the proteins responsible for cell motility (N, 2.77%), lipid transport and metabolism (I, 4.64%), and secondary metabolite biosynthesis, transport, and catabolism (Q, 3.25%) are significantly (P<0.001) higher than the average levels, which are 1.66%, 3.23%, and 1.86%, respectively. The abundances of the proteins responsible for carbohydrate transport and metabolism (G, 4.85%), defense mechanisms (V, 1.17%), and translation (J, 4.42%) are significantly (P<0.001) lower than the average levels, 6.69%, 1.79%, and 7.39%, respectively (Table S1).

One-sample *t* test was also made to evaluated the difference in gene abundance of difference COG categories between *Polymorphum gilvum* SL003B-26A1^T^ and other alkane degrading bacteria as well as the bacteria in IMG (Table S2). Indeed, *Polymorphum gilvum* SL003B-26A1^T^ was significantly (P<0.001) abundant in genes related to cell motility than other alkane degrading strains. Additionally, abundances of genes belonging to ‘carbohydrate transport and metabolism’ and ‘secondary metabolite biosynthesis, transport, and catabolism’ categories in *Polymorphum gilvum* SL003B-26A1^T^ were similar as those in other alkane degrading strains, but significantly (P<0.001) lower and higher than the corresponding average levels of all the bacteria genomes in the IMG. These results revealed a common feature in the alkane degrading bacteria, which is the low abundance of genes in carbohydrate metabolism and high abundance of genes in secondary metabolites biosynthesis.

#### Insertion sequences and gene transfer

Gene transfer, especially horizontal gene transfer (HGT), is a universally efficient way for microorganisms to acquire functions that enable them to adapt to environments with different selective pressures [36]–[38]. Genomic islands (GIs) in prokaryotic genomes are clusters of genes often regarded with horizontal origins [39]. There are 18 GIs predicted in the genome of the strain SL003B-26A1^T^ by SGI-HMM methods [40], [41] (Figure S2 and Table S3). In these 18 GIs, 119 CDS were identified, including CDS encoding regulators, ABC transport family systems, chaperone proteins for stress sensing, and oxidoreductases for metabolism (Table S4). For example, one gene island (659,055–667,832 bp) contains genes encoding acetate kinase (SL003B_0618, with a 65% maximum identity with that of *Agrobacterium radiobacter* K84), poly-beta-hydroxybutyrate polymerase for PHB synthesis from acetate (SL003B_0620, with a 74% maximum identity with that of *Paracoccus denitrificans* PD1222), and phosphate acetyl/butyryl transferase involved in the acetyl phosphate pathway in relation to the TCA cycle (SL003B_0619, with a 75% maximum identity with that of *Oligotropha carboxidovorans* OM5). A second gene island (678,054–690,058 bp) contains two sorts of genes: 1) genes encoding a LasR-LasI system (SL003B_0642 and SL003B_0643, with 56% and 47% maximum identities with those of *Collimonas fungivorans* Ter331 and *Mesorhizobium loti* MAFF303099, respectively) related to the regulation of biosurfactant synthesis, which is important for hydrocarbon emulsification and degradation; and 2) Hsp20 family proteins (SL003B_0651, with a 97% maximum identity with that of *Ochrobactrum anthropi* ATCC 49188) responsible for response to temperature changes to protect the cell from damage. Another gene island (745,551–753,026 bp) contains genes encoding proteins in a type I restriction modification system for responding to phage infection (SL003B_0710 and SL003B_0711, with 75% and 46% maximum identities with those of *Thalassiobium* sp. R2A62 and *Rhodopseudomonas palustris* TIE-1, respectively). The high identity of these predicted genes with those from different bacteria may suggest their potential origins from horizontal gene transfer, which are used to adapt to the alkane degradation, biosurfactant synthesis, and heat shock response in oil-contaminated saline soil with vibrating temperatures. Plenty of putative transposase and integrase coding genes, 63 CDS and 27 CDS, respectively, were identified in the chromosome and plasmid that could also support potential active HGT in the strain (Table S5).

#### Genomic comparisons with closely related bacteria

**Figure 2:**
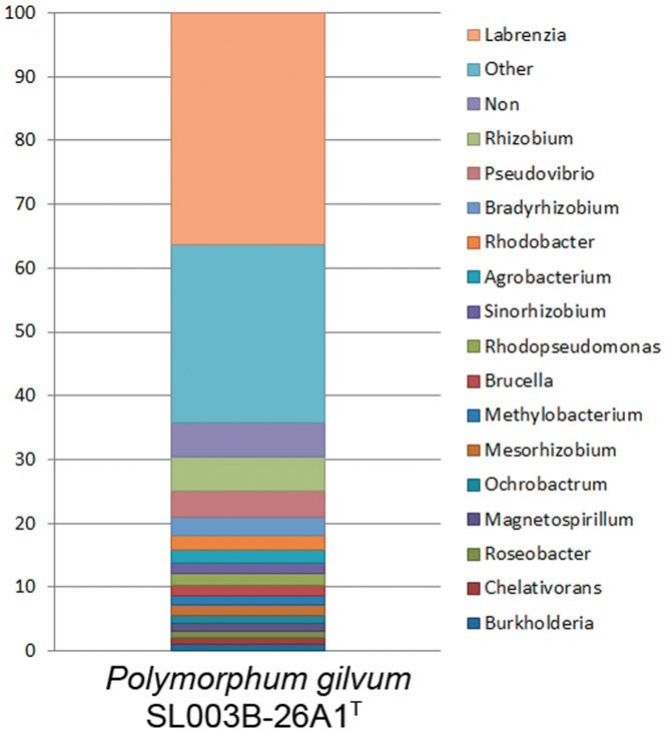
Taxonomic distribution analysis of the *Polymorphum gilvum* SL003B-26A1^T^ proteome.

The 16S rRNA gene sequence analyses revealed that *Polymorphum gilvum* SL003B-26A1^T^ is a new member of the family *Rhodobacteraceae*, closely related to but readily different from species in the genera of *Pannonibacter*, *Labrenzia*, *Roseibium*, and *Stappia* in the same *Rhodobacteraceae* family [34]. The taxonomic distribution analysis was performed by comparing each predicted protein in *Polymorphum gilvum* SL003B-26A1^T^ against all the proteins from the IMG microbial genome collection. The results revealed that the major proteins of *Polymorphum gilvum* SL003B-26A1^T^ are most closely matched to those in the genera *Labrenzia* (1571), *Rhizobium* (230), *Pseudovibrio* (177), *Bradyrhizobium* (122), *Rhodobacter* (99), and *Agrobacterium* (88) (Figure 2). Homologous comparisons between *Polymorphum gilvum* SL003B-26A1^T^ and the closest taxonomic distribution strains, *Labrenzia alexandrii* DFL-11, *Rhizobium rhizogenes* K84, *Pseudovibrio* sp. JE062, *Bradyrhizobium japonicum* USDA 110, *Rhodobacter sphaeroides* ATCC 17029, and *Agrobacterium* vitis S4, revealed the greatest overlap detected with *Labrenzia alexandrii* DFL-11. The whole genome MUMmer alignment also revealed that *Polymorphum gilvum* SL003B-26A1^T^ is closest to the genus *Labrenzia* because genome synteny is only found between *Polymorphum gilvum* SL003B-26A1^T^ and *Labrenzia alexandrii* DFL-11 with extensive rearrangement (Figure S1). However, the taxonomic distribution of the plasmid proteome is different from that of the chromosome. The majority of the proteins in the plasmid were most closely matched to bacteria belonging to *Agrobacterium*, *Rhizobium*, and *Nitrobacter*, suggesting the potentially different origins of the chromosome and the plasmid of the strain on the whole-genome scale.

#### Central metabolism

**Figure 3:**
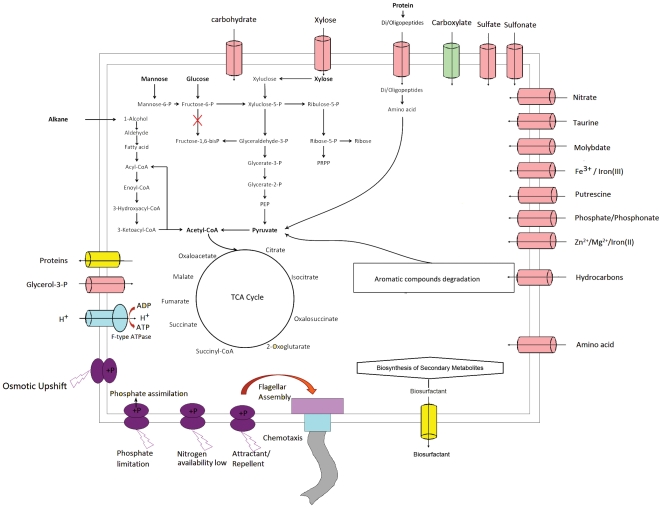
An overview of metabolism and transport in *Polymorphum gilvum* SL003B-26A1^T^.

Although SL003B-26A1^T^ could assimilate many sugars, including glucose, fructose, xylose, ribose, and mannose, the abundance of proteins in carbohydrate transport and metabolism category is lower than the average level in the IMG genome collection, i.e. 4.85% to 6.69%. Genes encoding all the enzymes needed in the glycolysis/gluconeogenesis pathway were found in the genome, except for fructose-6-phosphate kinase (EC: 2.7.1.11) (Figure 3), suggesting glucose cannot be converted to acetyl-CoA via the glycolysis pathway. Instead, the strain could use the pentose phosphate pathway (PPP) to convert glucose to glyceraldehyde-3-phosphate, and further to pyruvate and acetyl-CoA through the glycolysis pathway. In contrast, the alkane-degrading strains SK2, NG80-2, and AK-01 all have the complete glycolysis pathway through fructose-1, 6-phosphate, indicating the different carbohydrate metabolic pathway between strain SL003B-26A1T and strains SK2, NG80-2 and AK-01.

Strain SL003B-26A1^T^ contains all the enzymes for *de novo* amino acids synthesis and/or their interconversion. It also encodes 50 tRNAs for all 20 amino acids and one for selenocysteine, which decodes the codon UGA, commonly used as the stop codon in some organisms. Remarkably different from other oil-degrading bacteria, the strain harbors a complete selenocysteine synthesis pathway, and a selenate transport system.

Nine genes encoding the proposed fatty acid transporter (FAT) family found in the genome of strain SL003B-26A1^T^ indicate that the strain has a complete fatty acid metabolism pathway, which is important for alkanes degradation [14].

#### Regulation and transport

A total of 271 CDS in the genome were assigned to the transcription category (K) based on the COG analysis (Table S6), among which 32 proteins are LysR-type transcriptional regulators (LTTRs) (COG0583). This is the most abundant type of transcriptional regulator in the prokaryotic kingdom, especially throughout the different subdivisions of *proteobacteria* [42], which could play a regulatory role over genes involved in catabolism of aromatic compounds, cell motility, and quorum sensing. A further 21 proteins were found to belong to the multiple antibiotic resistance regulator (MarR) family transcriptional regulators (COG1846), responsible for bacterial response to antibiotics and catabolism of environmental aromatic compounds [43]; 19 proteins are TetR family transcriptional regulators (COG1309) and 27 proteins are response regulators consisting of a CheY-like receiver domain (COG0745, COG2197, COG3437, and COG4567) that form TCS with histidine kinases and are responsible for cell motility and bacteria chemotaxis [44]; 19 proteins are GntR family transcriptional regulators (COG1167, COG1802, COG2186, and COG2188) associated with the degradation of aromatic compounds [45]; and 13 proteins belong to AraC family transcriptional regulators (COG2207 and COG4977) responsible for sugar uptake and metabolism [46]. In addition, 11 proteins belong to AsnC family transcriptional regulators (COG 1522), (45), 6 proteins are IclR family transcriptional regulators (COG1414), 6 proteins are ArsR family transcriptional regulators (COG0640), 6 proteins are MerR family transcriptional regulators, and other proteins belong to the RpiR, LacI, and DeoR families.

Transport system analysis was performed by comparing each predicted protein against the Transport Classification Database (http://www.tcdb.org/) [47]. A total of 704 genes (16% of total CDS) involved in the transport system were found (Table S7). Among them, 313 genes were found to encode the ATP-binding Cassette (ABC) Superfamily (TC:3.A.1) related proteins that could import or export a broad range of compounds, such as carbohydrates, drugs, proteins, amino acids, inorganic anions, metal ions, lipids, and hydrocarbons; 77 genes encode proteins related to the tripartite ATP-independent periplasmic transporter (TRAP-T) family (TC: 2.A.56) for carboxylate transport; 22 genes encode proteins related to the Type IV (Conjugal DNA-Protein Transfer or VirB) Secretory Pathway (IVSP) Family (TC: 3.A.7) able to export proteins or DNA-protein complexes out of the cell and into the cytoplasm of a recipient cell; 20 genes encode proteins belonging to the Type III (Virulence-related) Secretory Pathway (IIISP) Family (TC: 3.A.6) that are often concerned with secretion of virulence factors. Another 272 genes were found to encode proteins related to 94 other transporter families, such as the Major Facilitator Superfamily (MFS) (TC: 2.A.1) for multidrug resistance and solute-cation (H^+^ or Na^+^) symport and/or solute-H^+^ or solute-solute antiport, the Drug/Metabolite Transporter (DMT) Superfamily (TC: 2.A.7) related to drug resistance and sugar transport, the H^+^- or Na^+^-translocating Bacterial Flagellar Motor/ExbBD Outer Membrane Transport Energizer (Mot-Exb) Superfamily (TC: 1.A.30) for bacterial flagellar rotation and accumulation of large molecules. Among these genes, two genes (SL003B_0097) encode proteins related to the K^+^ transporter (Trk) family (TC: 2.A.38) for K^+^ accumulation during osmotic shock, which might be associated with the adaptation of the strain in a high salinity environment.

### Genetic basis for crude oil degradation

#### Biosurfactant synthesis and crude oil emulsification

Crude oil components, such as hydrocarbons and aromatic compounds, are generally hydrophobic and of low availability to environmental microbes. Emulsification is a key step enabling bacteria to contact and degrade crude oil. Three categories of emulsification-related functions were found in the genome of SL003B-26A1^T^.

Firstly, biosurfactants, with glycolipids and lipopeptides reported as the most common ones produced by hydrocarbon degrading microbes [48], can emulsify and solublize the hydrocarbons to increase the microbial connection with oil components. Genes encoding the key enzymes in glycolipid synthesis in the genome of SL003B-26A1T, include acyltransferase, 3-oxoacyl-(acyl-carrier-protein) reductase, glycosyltransferase, phosphomannomutase (AlgC), and ketoreductase (RhlG) [49]. Three genes encoding OmpA-like proteins found are related to lipopeptide synthesis. The proteins are characteristic with its highly hydrophobic amino acid composition within four putative extra-membrane loops, which were suggested to be the active component of the bioemulsifier alasan [50], [51] (Table S8).

At the regulation level, 12 LuxR regulator coding genes were identified, including two LasR-LasI TCS systems (SL003B_0642-0643 and SL003B_0701-0702). The LasR regulator was reported to be related to regulation of glycolipid biosynthesis under the autoinducer LasI [52].

Thirdly, strain SL003B-26A1^T^ has 12 genes for type VI pili assembly, which mediates biofilm formation and microbial adhesion to biotic and abiotic surfaces [53], [54], and the oil-water interface. The TCS for flagella biosynthesis and cell motility was also identified, including the response regulators CheY (Table S11 and Table S12) activated by the regulators CheA (SL003B_0948), CheW (SL003B_0949), CheB (SL003B_0951), and CheR (SL003B_0952) [55]. Flagella assembly plays an important role in cell motility and chemotaxis, which could also help bacteria move to relatively better niches and attach to the oil-water interface where the degradation of alkanes can take place and more carbon sources are available for growth (Table S12 and Table S13).

In summary, the genes responsible for glycolipids and lipopeptides synthesis as well as pili and flagella assembly are well quipped in the strain which can function to emulsify the hydrocarbons for degradation of crude oil.

#### Degradation of crude oil components

**Figure 4:**
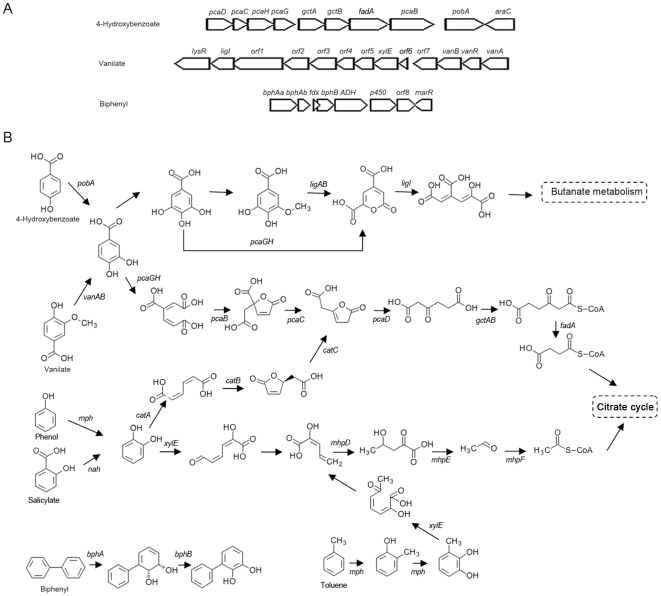
Gene clusters involved in aromatic compounds metabolisms in SL003B-26A1 and putative metabolic pathways. A: Gene clusters involved in aromatic compounds metabolisms. B: Putative pathways of aromatic compounds metabolisms. *pcaD*, 3-oxoadipate enol-lactonase; *pcaC*, 4-carboxymuconolactone decarboxylase; *pcaH*, protocatechuate 3,4-dioxygenase, beta subunit; *pcaG*, protocatechuate 3,4-dioxygenase, alpha subunit; *gctA*, glutaconate CoA-transferase, subunit A; *gctB*, glutaconate CoA-transferase, subunit B; *fadA*, acetyl-CoA acyltransferase; *pcaB*, 3-carboxy-cis,cis-muconate cycloisomerase; *pobA*, p-hydroxybenzoate 3-monooxygenase; *araC*, AraC-type DNA-binding domain-containing proteins; *lysR*, putative LysR family transcriptional regulator; *ligI*, 2-pyrone-4,6-dicarboxylate lactonase; *orf*1, TRAP-type uncharacterized transport system, fused permease components; *orf*2, TRAP-type uncharacterized transport system, periplasmic component; *orf*3, hypothetical protein; *orf*4, demethylmenaquinone methyltransferase; *orf*5, GlcNAc-PI de-N-acetylase family protein; *xylE*, catechol 2,3-dioxygenase; *orf*6, hypothetical protein; *orf*7, 3-hydroxyisobutyrate dehydrogenase and related beta-hydroxyacid dehydrogenases; *vanB*, vanillate monooxygenase, subunit B; *vanR*, GntR family transcriptional regulator, vanillate catabolism transcriptional regulator; *vanA*, vanillate monooxygenase, subunit A; *bphAa*, biphenyl 2,3-dioxygenase, large subunit; *bphAb*, biphenyl 2,3-dioxygenase, small subunit; *fdx*, ferredoxin; *bphB*, cis-2,3-dihydrobiphenyl-2,3-diol dehydrogenase; *ADH*, aldehyde dehydrogenase; *p450*, cytochrome P450 family protein; *orf*8, 2-keto-4-pentenoate hydratase/2-oxohepta-3-ene-1,7-dioic acid hydratase; *marR*, MarR family transcription regulator protein; *ligA*, protocatechuate 4,5-dioxygenase, alpha chain; *ligB*, protocatechuate 4,5-dioxygenase, beta chain; *catA*, catechol 1,2-dioxygenase; *catB*, muconate cycloisomerase; *catC*, muconolactone D-isomerase; *mph*, phenol 2-monooxygenase; *nah*, salicylate 1-monooxygenase; mhpD, 2-keto-4-pentenoate hydratase; *mhpE*, 4-hydroxy 2-oxovalerate aldolase; *mhpF*, acetaldehyde dehydrogenase.

Genes corresponding to the degradation of oil components, including aromatic compounds and alkanes, were identified in the genome of SL003B-26A1^T^. For aromatic compound degradation, gene could include: benzoyl CoA synthetase (SL003B_1861) in benzoate degradation; phenol 2-monooxygenase (SL003_1806) in toluene and resorcinol degradation; aromatic-ring-hydroxylating dioxygenase (SL003B_4095, SL003B_4096) in naphthalene, ethylbenzene, biphenyl, and chlorobiphenyl degradation; and catechol 1,2-dioxygenase (SL003B_1105, SL003B_3188) and catechol 2,3-dioxygenase (SL003B_2858, SL003B_4080, and SL003B_4107) in catechol degradation. There are also numerous oxidoreductases, hydroxylases, dehydrogenases, and dioxygenases related to the degradation of PAHs, cyclic hydrocarbons, and other aromatic compounds (Figure 4 and Table S9).

Strikingly, strain SL003B-26A1^T^ contains more genes related to the degradation of aromatic compounds than other known oil-degrading bacteria. For example, genes encoding catechol 1,2-dioxygenase, catechol 2,3-dioxygenase, vanillate monooxygenase, phenol 2-monooxygenase, and salicylate hydroxylase are only found in the genome of SL003B-26A1^T^, and genes encoding benzoyl CoA synthetase are only found in the genomes of SL003B-26A1^T^ and NG80-2 but are not found in the marine bacteria Sk-2 and Ak-01. The high abundance of aromatic-compound-degrading genes in SL003B-26A1^T^ might be a marked characteristic of this bacterium derived from an oil-contaminated environment, which is in accord with a previous finding that aromatic hydrocarbon degradation genes, such as monooxygenase and dioxygenase, were detected in high abundance in oil-contaminated fields [56].

At the regulation level, several LTTRs were found as putative regulators of genes related to the catabolism of aromatic compounds in the genome of SL003B-26A1^T^, including LTTR (SL003B_1862) regulating gene encoding benzoyl CoA synthetase (SL003B_1861) and LTTR (SL003B_1103) regulating genes encoding maleylacetate reductase (SL003B_1104) and catechol 1,2-dioxygenase (SL003B_1105). In addition, a gene encoding putative MarR family regulator (SL003B_4102) is related to biphenyl degradation gene regulation (SL003B_4095-SL003B_4098). GntR family transcriptional regulators found in the strain are associated with aromatic compound degradation [45], including the putative GntR regulator (SL003B_3187) related to the regulation of catechol 1,2-dioxygenase genes (SL003B_3188) and regulator (SL003B_2862) related to the regulation of the catechol 2,3-dioxygenase gene (SL003B_2858) in catechol catabolism.

Furthermore, genes for transferring the intermediary metabolites from aromatic compound metabolism into the central metabolism were also predicted in the genome, indicating the presence of complete pathways for aromatic compound degradation and metabolism. These genes found in the genome also support the genetic basis of *Polymorphum gilvum* SL003B-26A1^T^ for using aromatic oil components as carbon sources.

As for alkane degradation, no AlkB homolog coding genes were found, but a long alkane hydroxylase (LadA) coding gene (SL003B_1417, with 36% identity with LadA in NG80-2) [57] was found in SL003B-26A1^T^. The presence of an alkane hydroxylase and an alcohol dehydrogenase and an aldehyde dehydrogenase necessary for alkane degradation as well as fatty acid metabolism genes suggest a complete alkane degradation pathway in SL003B-26A1^T^. It was different that the gene coding for LadA was located on the plasmid of strain NG80-2, but on the chromosome in SL003B-26A1T.

### Genetic basis for response to a saline environment

Environmental bacteria can evolve systems for adapting to environments in which they are living, including sensing and responding systems, regulation systems, and systems of metabolism, transportation, and so on. Diverse and abundant genes in these systems may suggest the strong ability of the cells to adapt to their living environments.

Based on the COG analysis, 197 genes in the genome were assigned to the signal transduction category (Table S10). Among them, 35 genes were predicted to encode kinases and 52 genes were predicted to encode putative response regulators. The TCS are a basic stimulus-response coupling mechanism in bacteria for sensing and responding to changes in many different environmental conditions. In the SL003B-26A1^T^ genome, 20 complete TCS were identified and predicted to sense and respond to phosphate limitation, osmolarity, C4-dicarboxylate, nitrogen concentration, and other attractants or repellents in the niche (Table S11).

At the regulation level, the TCS responsible for flagella biosynthesis and cell motility might be essential for cells living in a harsh environment [58]. The genes for regulation of flagella assembly were also found in the strains NG80-2 and AK-01, but were not found in strain SK2. The fact that the abundance of genes associated with cell motility in the SL003B-26A1^T^ genome is above the average level (2.77% vs. 1.66%) could be the result of this strain adapting to oil-polluted saline soil (Table S12). The TCS for osmotic responses (EnvZ/OmpR) [22], [59] and heat shock [31] were also identified, which could explain why strain SL003B-26A1^T^ can survive in a saline environment at a temperature of 45°C. The EnvZ protein can exist in two alternative conformational states, a high osmolarity form and a low osmolarity form. In the high osmolarity niche, EnvZ is activated and transfers a phosphoryl group to OmpR. In the low osmolarity niche, EnvZ exhibits a lower kinase activity but its stimulation of OmpR dephosphorylation is enhanced [60]. Other TCS, KdpD/KdpE [61] and MtrA/MtrB [62], for responses to osmotic stress were not found. The same EnvZ/OmpR system was also found in the marine strain SK2. No TCS for osmotic stress response were found in the strains NG80-2 and AK-01. In addition, genes encoding regulators consisting of a CheY-like receiver domain were identified with putative functions in response to phosphate limitation (SL003B_3275) and osmolarity stress (SL003B_1203). Heat shock proteins belonging to the Hsp90, Hsp20, and Hsp33 families were identified, which should be responsible for regulating the expression of heat response proteases such as ATP-dependent metalloprotease FtsH (SL003B_0653, SL003B_0928), ATP-dependent Clp protease (SL003B_1811, SL003B_1812, SL003B_1826, SL003B_1827, SL003B_2063, and SL003B_2064), and ATP-dependent protease HslVU (SL003B_4321 and SL003B_4322). The strain SL003B-26A1^T^ also contains cold shock genes encoding Csp [63] (SL003B_1226, SL003B_1984, SL003B_3547, SL003B_3721, and SL003B_4222) for cold stress response and regulation, which could also reflect the surroundings when hot production water was not discharged in day time of winter when it is frozen.

After regulation, various functions can be expressed. For example, the most rapid response to counteract osmotic upshift is stimulation of K^+^ uptake and then the accumulation of potassium glutamate [28]–[30] by genes related to K^+^ uptake systems (SL003B_0097, SL003B_1618 and SL003B_2301). In addition to K^+^ uptake systems, genes linking the responses for osmotic stress and nitrogen limitation recently found in *Escherichia coli* [64] were also found in strain SL003B-26A1^T^. GlnL/GlnG (NtrB/NtrC) and NtrY/NtrX TCS are reported to be involved in nitrogen regulation and glutamate assimilation [65]–[67]. In the strain SL003B-26A1, a gene encoding Trk system potassium uptake protein (TrKA) (SL003B_2301), which could uptake K^+^, is located immediately downstream of the *ntrY*-*ntrX* and *glnL*-*glnG* operon and might be regulated under available nitrogen limitation, such as growth with ammonium as the sole nitrogen source. As for heat shock protection, the expression of chaperone proteins DnaJ (SL003B_0343, SL003B_1332, SL003B_1823, and SL003B_4325), DnaK (SL003B_3893 and SL003B_4327) and GrpE (SL003B_4194) and repressor protein HrcA (SL003B_0022) can result in refolding or removal of heat damaged proteins.

### Comparisons among oil-degrading bacteria

Analyses of the abundance of the COG categories of *Alcanivorax borkumensis* SK2, *Geobacillus thermodenitrificans* NG80-2, and *Desulfatibacillum alkenivorans* AK-01 revealed similarities and differences among these hydrocarbon-degrading strains. The toxicity and low availability of oil components as carbon sources could be the driving forces for these bacteria to evolve sensitive sensing and response systems to avoid damage by hydrocarbons and pursue nutrients. In the genomes of SL003B-26A1^T^, SK2, NG80-2, and AK-01, the abundances of protein categories responsible for carbohydrate transport and metabolism are lower than the average value for all genomes in the IMG database. This is in accord with the low carbohydrate availability in the environments where these strains were isolated. Fatty acids are important intermediate products in the alkane degradation pathway, and lipid transport and metabolism are therefore important for the further degradation of alkanes. It is therefore reasonable that the abundances of protein categories responsible for lipid transport and metabolism, and secondary metabolite biosynthesis, transport, and catabolism, are higher in the genomes of SL003B-26A1^T^, SK2, NG80-2, and AK-01 than the average level in all other genomes. The high abundance of lipid metabolism-related proteins in these strains reveals the genetic basis of the conversion of alkanes to energy. The biosurfactants synthesized as secondary metabolites are also essential in crude oil degradation, in which emulsification of the crude oil could help the strains to utilize hydrocarbons more easily [68], [52]. Furthermore, it is interesting that although the strain SK2, isolated from marine sediment, could utilize crude oil, proteins in SK2 belonging to cell motility COG categories are fewer than those in SL003B-26A1^T^ and NG80-2, which were isolated from terrestrial oil-contaminated environments. The reason why cell motility is more important for SL003B-26A1^T^ and NG80-2 may be that soil and oil reservoirs are much more compacted.

### Conclusions

*Polymorphum gilvum* SL003B-26A1^T^ was isolated from an oil-polluted environment and could utilize numerous compounds derived from oil, such as alkanes and aromatic hydrocarbons, as its sole carbon sources. The genome of SL003B-26A1^T^ reported here provides the genetic basis of a bacterial lifestyle in an oil-contaminated environment. Genes involved in hydrocarbon degradation, environment stress sensing and response, signal transduction, cell defenses, and HGT were identified in its genome, and point to the unique abilities of SL003B-26A1^T^ in oil degradation and extreme environment adaptation. Genomic research on SL003B-26A1^T^ would also provide a blueprint for the application of bioremediation in oil-polluted environments and microbial-enhanced oil recovery.

## Materials and Methods

### Strain and culture conditions

The strain *Polymorphum gilvum* SL003B-26A1^T^ was isolated from crude oil-polluted soil in Shengli Oilfield, eastern China [34]. The soil was saline with a dissolved salt content of 3.8 mS·cm^−1^. The sampling point was a site to discharge the treated oil-production wastewater with a temperature of ca. 45°C all the year long. When wastewater was discharged in the night, the sampling site was heated to ca. 45°C, however, in daytime in winter when the wastewater was not discharged then site could be frozen. After the cells of SL003B-26A1^T^ were grown in Lysogeny Broth (LB) medium at 30°C for 3 days, genomic DNA was isolated [69]. To examine growth on crude oil components, SL003B-26A1^T^ was grown in a minimal medium (5 g NaCl, 1 g NH_4_H_2_PO_4_, 1 g (NH_4_)_2_SO_4_, 1 g K_2_HPO_4_, 0.2 g MgSO_4_, and 3 g KNO_3_ per liter deionized water, pH = 7.2) supplemented with 0.1% (vol/vol) MT microelements (MT stock contains 2.78 g of FeSO_4_·7H_2_0, 1.98 g of MnCl_2_·4H_2_0, 2.81 g of CoS0_4_·7H_2_0, 1.47 g of CaCl_2_·2H_2_0, 0.17 g of CuCl_2_·2H_2_0, and 0.29 g of ZnSO_4_·7H_2_0 in 1 N HCl per liter deionized water) and 0.1% (wt/vol) crude oil from Shengli Oilfield, China [70] as the sole carbon source.

### Genome sequencing

The complete genome sequencing of *Polymorphum gilvum* SL003B-26A1^T^ was performed with a combined strategy of 454 sequencing [71] and Solexa paired-end sequencing [72] technologies. Genomic libraries containing 8-kb inserts were constructed. A total of 248,467 paired-end reads were generated using the GS FLX system (454 Life Sciences Corporation, Branford, CT), giving a 64.0-fold coverage of the genome. And 96.4% of the reads were assembled into two large scaffolds by using the 454 Newbler assembler, including 139 nonredundant contigs. A total of 3,487,313 reads (3-kb library) were generated with an Illumina Solexa Genome Analyzer IIx (Illumina, San Diego, CA) to reach a depth of 151.5-fold coverage and mapped to the scaffolds using the Burrows-Wheeler Alignment (BWA) tool [73]. The gaps between the scaffolds were filled by sequencing PCR products using an ABI 3730 capillary sequencer.

### Genome analysis

Protein encoding genes were predicted by Glimmer 3.0 [74]. The analysis of the genome was performed as described previously [35], [75]. Genomic islands (GIs) were analyzed using IslandViewer (http://www.pathogenomics.sfu.ca/islandviewer) [76]. The genome sequence was also submitted to the Integrated Microbial Genomes (IMG) server (http://img.jgi.doe.gov) of the Joint Genome Institute (JGI) for deep analysis and genome comparison [77]. A one-sample *t* test was used to evaluate the statistically significant differences of gene abundance in each COG category between *Polymorphum gilvum* SL003B-26A1^T^ and other genomes deposited in the IMG bacteria genome database. A total of 2,634 bacteria genomes were selected for COG analysis.

### Nucleotide sequence accession number

The nucleotide sequence of *Polymorphum gilvum* SL003B-26A1^T^ has been deposited in the GenBank database under accession numbers CP002568 (chromosome) and CP002569 (plasmid).

## Supporting Information

Figure S1 **Synteny plots between Polymorphum gilvum SL003B-26A1T genome (X axis) and other closely related genomes.** *Labrenzia alexandrii* DFL-11.(A), *Rhizobium rhizogenes* K84 (B), *Pseudovibrio sp.* JE062 (C), *Bradyrhizobium japonicum* USDA 110 (D), *Rhodobacter sphaeroides* ATCC 17029 (E), *Agrobacterium vitis* S4 (F), by Mummer using protein sequence based comparisons. Red = leading strand; blue = lagging strand.
(TIFF)

Figure S2 **Genomic Islands (GIs) prediction by different methods.** Ring 1 (red) (from outside in) indicates the GIs by multiple methods; ring 2 (blue) indicated the GIs predicted by IslandPath-DIMOB method; ring 3 (orange) indicated the GIs predicted by SIGI-HMM method; the black line plot indicates the G+C content.
(TIFF)

Table S1 **Comparative analysis of COG categories between** ***Polymorphum gilvum*** **SL003B-26A1^T^ and other selected bacteria genomes in IMG bacteria genome database.**
(DOC)

Table S2 **Comparative analysis of COG categories between Polymorphum gilvum SL003B-26A1T and other genomes of alkane degrading bacteria in IMG bacteria genome database.**
(DOC)

Table S3 **Genomic islands prediction by different methods.**
(DOC)

Table S4 **Genes in predicted GIs by SIGI-HMM program.**
(DOC)

Table S5 **Insertion sequences predicted in SL003B-26A1^T^.**
(DOC)

Table S6 **Transcription (COG category K).**
(DOC)

Table S7 **Transporters.**
(DOC)

Table S8 **Genes in biosurfactant synthesis.**
(DOC)

Table S9 **Genes in hydrocarbon and aromatic compounds degradation.**
(DOC)

Table S10 **Genes in signal transduction (COG category T).**
(DOC)

Table S11 **Genes in two component systems (pathways via KO terms).**
(DOC)

Table S12 **Genes in cell motility (COG category N).**
(DOC)

Table S13 **Genes in cell motility and Chemotaxis (pathways via KO terms).**
(DOC)
