# Supplementary figures and images for: The Genome Sequence of Polymorphum gilvum SL003B-26A1T Reveals Its Genetic Basis for Crude Oil Degradation and Adaptation to the Saline Soil
Source: PLoS One. 2012 Feb 16;7(2):e31261. doi: 10.1371/journal.pone.0031261 (PMC3281065; doi:10.1371/journal.pone.0031261)

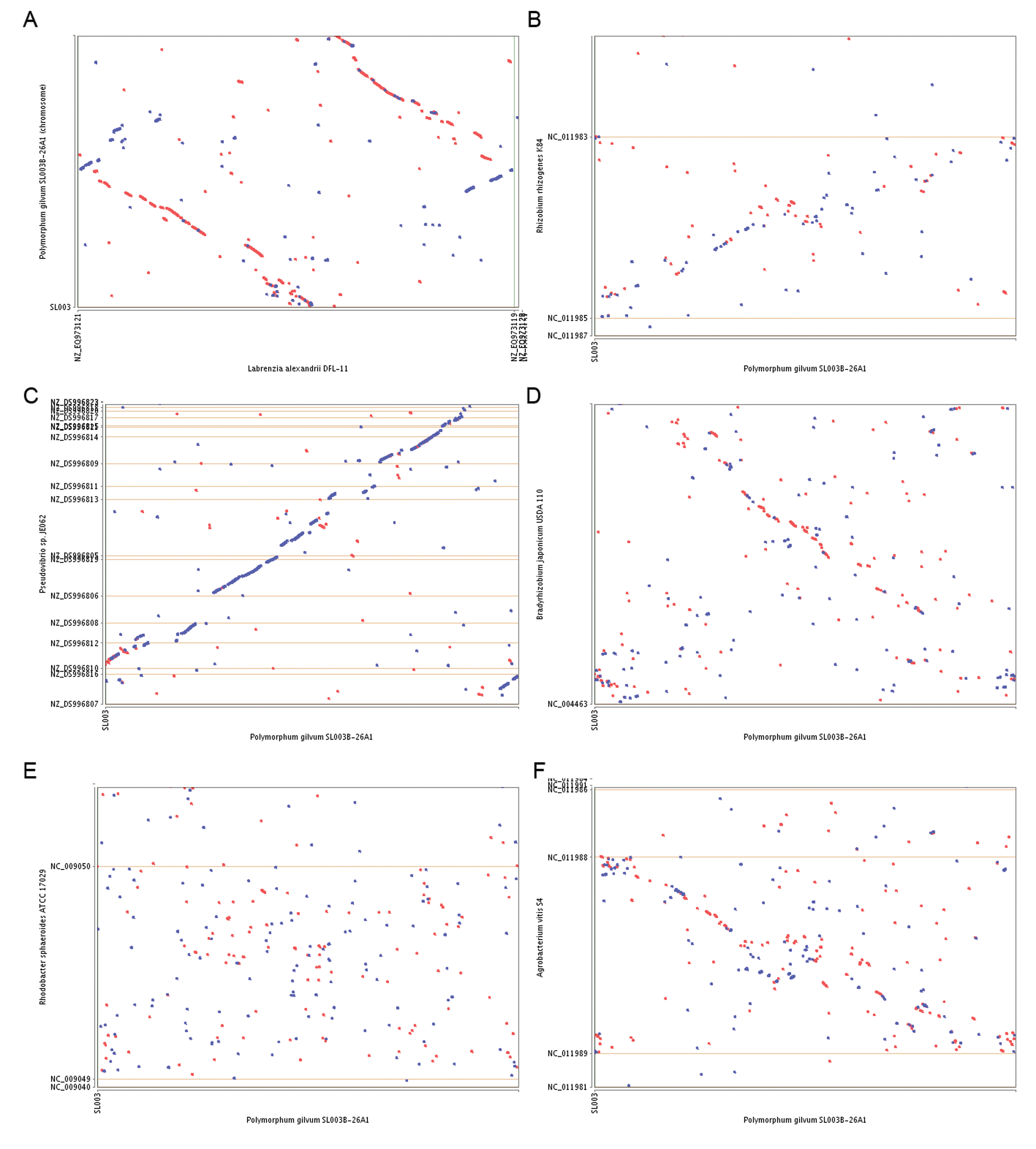

Supplement: Figure S1 — Synteny plots between Polymorphum gilvum SL003B-26A1T genome (X axis) and other closely related genomes. Labrenzia alexandrii DFL-11.(A), Rhizobium rhizogenes K84 (B), Pseudovibrio sp. JE062 (C), Bradyrhizobium japonicum USDA 110 (D), Rhodobacter sphaeroides ATCC 17029 (E), Agrobacterium vitis S4 (F), by Mummer using protein sequence based comparisons. Red = leading strand; blue = lagging strand. (TIFF) [file pone.0031261.s001.tif]

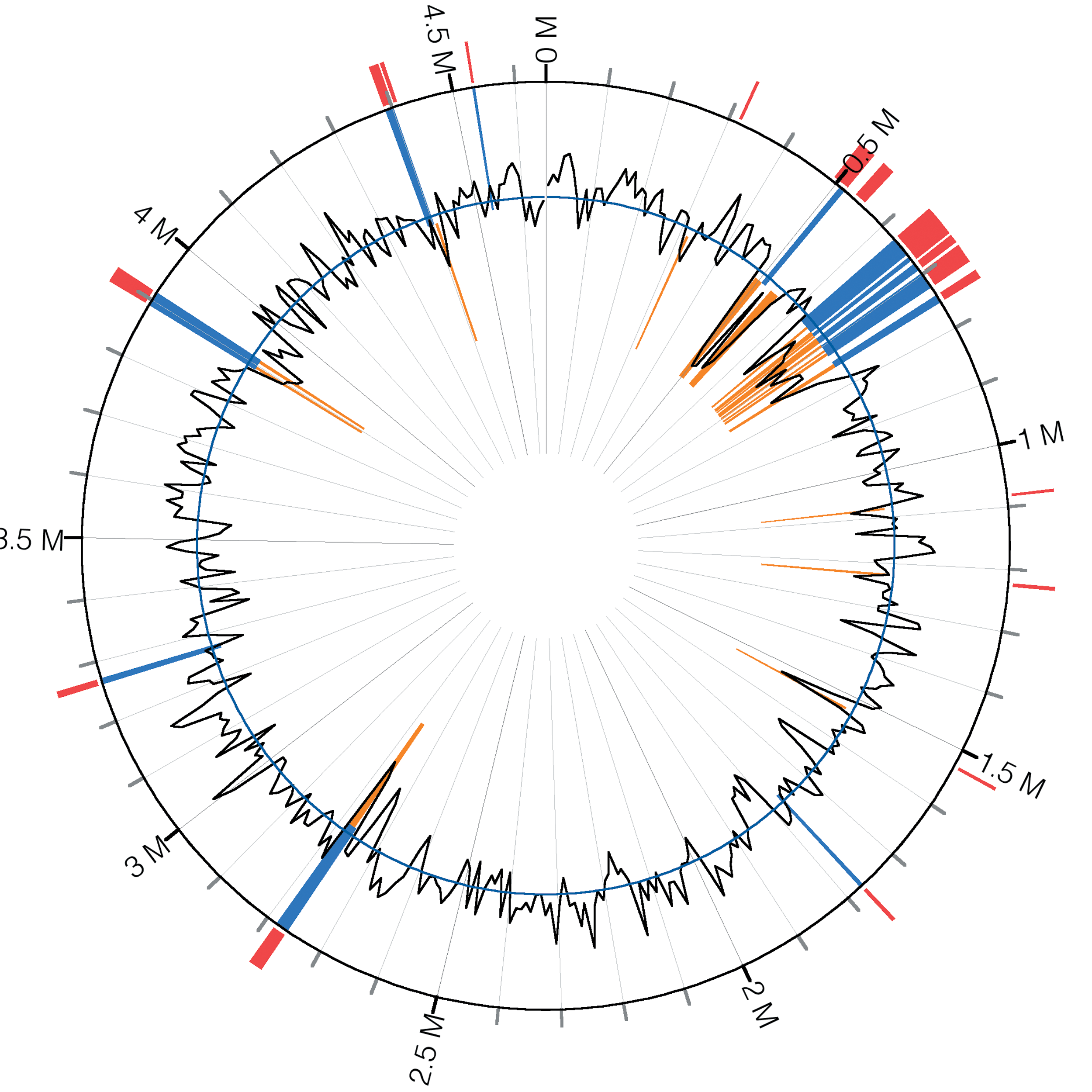

Supplement: Figure S2 — Genomic Islands (GIs) prediction by different methods. Ring 1 (red) (from outside in) indicates the GIs by multiple methods; ring 2 (blue) indicated the GIs predicted by IslandPath-DIMOB method; ring 3 (orange) indicated the GIs predicted by SIGI-HMM method; the black line plot indicates the G+C content. (TIFF) [file pone.0031261.s002.tif]
