# Supplementary material for: The Genome Sequence of Polymorphum gilvum SL003B-26A1T Reveals Its Genetic Basis for Crude Oil Degradation and Adaptation to the Saline Soil
Source: PLoS One. 2012 Feb 16;7(2):e31261. doi: 10.1371/journal.pone.0031261 (PMC3281065; doi:10.1371/journal.pone.0031261)
Supplement: Table S1 — Comparative analysis of COG categories between Polymorphum gilvum SL003B-26A1T and other selected bacteria genomes in IMG bacteria genome database. (DOC) [file pone.0031261.s003.doc]

## Table S1 Comparative analysis of COG categories between *Polymorphum gilvum* SL003B-26A1^T^ and other selected genomes in IMG bacteria genome database.

| COG category | Gene Abundance (%) | | Std. Deviation (%) | Std. Error Mean (%) | *t*-score | *P*-value (2-tailed) |
| --- | --- | --- | --- | --- | --- | --- |
|  | 26A1 | Mean |  |  |  |  |
| Amino acid transport and metabolism | 10.35 | 8.23 | 1.97 | 0.038 | -52.809 | 0.000 |
| Carbohydrate transport and metabolism | 4.85 | 6.69 | 2.75 | 0.053 | 34.425 | 9.3E-215 |
| Cell cycle control, cell division, chromosome partitioning | 0.89 | 1.26 | 0.64 | 0.012 | 29.592 | 2.2E-166 |
| Cell motility | 2.77 | 1.66 | 1.50 | 0.029 | -37.7 | 4.2E-249 |
| Cell wall/membrane/envelope biogenesis | 4.87 | 5.78 | 1.72 | 0.034 | 26.971 | 1.1E-141 |
| Coenzyme transport and metabolism | 3.91 | 4.47 | 1.27 | 0..025 | 22.89 | 6.6E-106 |
| Defense mechanisms | 1.17 | 1.79 | 0.82 | 0.016 | 38.835 | 3.1E-261 |
| Energy production and conversion | 6.45 | 5.86 | 1.58 | 0.031 | -19.129 | 1.8E-76 |
| Function unknown | 9.39 | 8.23 | 2.03 | 0.04 | -29.293 | 1.6E-163 |
| General function prediction only | 11.72 | 11.49 | 2.64 | 0.051 | -4.567 | 5.2E-6 |
| Inorganic ion transport and metabolism | 5.56 | 5.05 | 1.16 | 0.023 | -22.315 | 3.6E-101 |
| Intracellular trafficking, secretion, and vesicular transport | 2.72 | 2.40 | 1.18 | 0.023 | -13.903 | 1.8E-42 |
| Lipid transport and metabolism | 4.64 | 3.23 | 1.41 | 0.028 | -51.464 | 0.000 |
| Nucleotide transport and metabolism | 2.08 | 2.93 | 0.98 | 0.019 | 44.532 | 0.000 |
| Posttranslational modification, protein turnover, chaperones | 3.83 | 3.90 | 1.17 | 0.023 | 2.818 | 0.005 |
| Replication, recombination and repair | 5.18 | 6.19 | 2.23 | 0.046 | 22.088 | 2.5E-99 |
| RNA processing and modification | 0.025 | 0.051 | 1.17 | 0.003 | 7.631 | 0.025 |
| Signal transduction mechanisms | 5.00 | 4.51 | 2.11 | 0.041 | -11.952 | 4.2E-32 |
| Secondary metabolites biosynthesis, transport and catabolism | 3.25 | 1.86 | 1.37 | 0.027 | -51.911 | 0.000 |
| Transcription | 6.88 | 6.81 | 2.10 | 0.041 | -1.607 | 0.108 |
| Translation, ribosomal structure and biogenesis | 4.42 | 7.39 | 4.03 | 0.079 | 37.822 | 2.1E-250 |
