# Supplementary material for: The Genome Sequence of Polymorphum gilvum SL003B-26A1T Reveals Its Genetic Basis for Crude Oil Degradation and Adaptation to the Saline Soil
Source: PLoS One. 2012 Feb 16;7(2):e31261. doi: 10.1371/journal.pone.0031261 (PMC3281065; doi:10.1371/journal.pone.0031261)
Supplement: Table S2 — Comparative analysis of COG categories between Polymorphum gilvum SL003B-26A1T and other genomes of alkane degrading bacteria in IMG bacteria genome database. (DOC) [file pone.0031261.s004.doc]

## Table S2 Comparative analysis of COG categories between *Polymorphum gilvum* SL003B-26A1^T^ and other genomes of alkane degrading bacteria in IMG bacteria genome database

| COG category | Gene Abundance (%) | | Std. Deviation (%) | Std. Error Mean (%) | *t*-score | *P*-value (2-tailed) |
| --- | --- | --- | --- | --- | --- | --- |
|  | 26A1 | Mean |  |  |  |  |
| Amino acid transport and metabolism | 10.35 | 8.62 | 1.64 | 0.38 | -4.606 | 2.2E-4 |
| Carbohydrate transport and metabolism | 4.85 | 4.88 | 1.32 | 0.30 | 0.119 | 0.907 |
| Cell cycle control, cell division, chromosome partitioning | 0.89 | 0.98 | 0.29 | 0.07 | 1.388 | 0.182 |
| Cell motility | 2.77 | 1.69 | 1.1 | 0.25 | -4.271 | 4.6E-4 |
| Cell wall/membrane/envelope biogenesis | 4.87 | 5.06 | 1.31 | 0.30 | 0.64 | 0.530 |
| Coenzyme transport and metabolism | 3.91 | 4.41 | 0.50 | 0.12 | 4.397 | 3.5E-4 |
| Defense mechanisms | 1.17 | 1.40 | 0.33 | 0.08 | 3.001 | 0.008 |
| Energy production and conversion | 6.45 | 6.78 | 1.22 | 0.28 | 1.190 | 0.250 |
| Function unknown | 9.39 | 8.48 | 1.22 | 0.28 | -3.258 | 0.004 |
| General function prediction only | 11.72 | 12.40 | 1.05 | 0.24 | 2.803 | 0.012 |
| Inorganic ion transport and metabolism | 5.56 | 5.18 | 1.09 | 0.25 | -1.492 | 0.153 |
| Intracellular trafficking, secretion, and vesicular transport | 2.72 | 2.33 | 1.35 | 0.31 | -1.243 | 0.230 |
| Lipid transport and metabolism | 4.64 | 5.64 | 1.79 | 0.41 | 2.419 | 0.026 |
| Nucleotide transport and metabolism | 2.08 | 2.21 | 0.42 | 0.10 | 1.369 | 0.188 |
| Posttranslational modification, protein turnover, chaperones | 3.83 | 3.47 | 0.74 | 0.17 | -2.103 | 0.050 |
| Replication, recombination and repair | 5.18 | 4.59 | 1.30 | 0.30 | -1.966 | 0.065 |
| RNA processing and modification | 0.025 | 0.03 | 0.03 | 0.01 | 0.998 | 0.332 |
| Signal transduction mechanisms | 5.00 | 5.17 | 1.91 | 0.44 | 0.390 | 0.701 |
| Secondary metabolites biosynthesis, transport and catabolism | 3.25 | 3.63 | 1.74 | 0.40 | 0.969 | 0.345 |
| Transcription | 6.88 | 8.14 | 2.02 | 0.46 | 2.716 | 0.014 |
| Translation, ribosomal structure and biogenesis | 4.42 | 4.83 | 1.29 | 0.30 | 1.406 | 0.177 |

(The genomes of *Acinetobacter baylyi* ADP1, *Acinetobacter lwoffii* SH145, *Alcanivorax borkumensis* SK2, *Bacillus thuringiensis* Bt407, *Burkholderia cepacia* 383, *Geobacillus thermodenitrificans* NG80-2, *Gordonia bronchialis* DSM 43247, *Pseudomonas aeruginosa* PAO1, *Pseudomonas fluorescens* Pf-5, *Rhodococcus jostii* RHA1, *Desulfococcus oleovorans* Hxd3, *Desulfatibacillum alkenivorans* AK-01, *Marinobacter algicola* DG893, *Mycobacterium bovis* AF2122/97, *Nocardia farcinica* IFM 10152, *Paracoccus denitrificans* PD1222 and *Xylella fastidiosa* 9a5c were selected as the candidates of alkane degrading bacteria for COG analysis)
