# Supplementary material for: The Genome Sequence of Polymorphum gilvum SL003B-26A1T Reveals Its Genetic Basis for Crude Oil Degradation and Adaptation to the Saline Soil
Source: PLoS One. 2012 Feb 16;7(2):e31261. doi: 10.1371/journal.pone.0031261 (PMC3281065; doi:10.1371/journal.pone.0031261)
Supplement: Table S3 — Genomic islands prediction by different methods. (DOC) [file pone.0031261.s005.doc]

## Table S3 Genomic islands prediction by different methods

| **Start** | **End** | **Size** | **GI Prediction Program** |
| --- | --- | --- | --- |
| 314738 | 318897 | 4159 | Predicted by multiple methods |
| 491075 | 517135 | 26060 | Predicted by multiple methods |
| 535070 | 553665 | 18595 | Predicted by multiple methods |
| 628016 | 674105 | 46089 | Predicted by multiple methods |
| 678054 | 690692 | 12638 | Predicted by multiple methods |
| 695587 | 724924 | 29337 | Predicted by multiple methods |
| 739692 | 753026 | 13334 | Predicted by multiple methods |
| 1079267 | 1083438 | 4171 | Predicted by multiple methods |
| 1221849 | 1227436 | 5587 | Predicted by multiple methods |
| 1526662 | 1531587 | 4925 | Predicted by multiple methods |
| 1767658 | 1773351 | 5693 | Predicted by multiple methods |
| 2762572 | 2783419 | 20847 | Predicted by multiple methods |
| 3263421 | 3273176 | 9755 | Predicted by multiple methods |
| 3890826 | 3914156 | 23330 | Predicted by multiple methods |
| 4387588 | 4401246 | 13658 | Predicted by multiple methods |
| 4404522 | 4409716 | 5194 | Predicted by multiple methods |
| 4531507 | 4535124 | 3617 | Predicted by multiple methods |
| 314738 | 318897 | 4159 | SIGI-HMM |
| 491075 | 507821 | 16746 | SIGI-HMM |
| 535070 | 553665 | 18595 | SIGI-HMM |
| 644585 | 649481 | 4896 | SIGI-HMM |
| 659055 | 667832 | 8777 | SIGI-HMM |
| 670085 | 674105 | 4020 | SIGI-HMM |
| 678054 | 690058 | 12004 | SIGI-HMM |
| 695587 | 701085 | 5498 | SIGI-HMM |
| 708233 | 712721 | 4488 | SIGI-HMM |
| 716282 | 721440 | 5158 | SIGI-HMM |
| 745551 | 753026 | 7475 | SIGI-HMM |
| 1079267 | 1083438 | 4171 | SIGI-HMM |
| 1221849 | 1227436 | 5587 | SIGI-HMM |
| 1526662 | 1531587 | 4925 | SIGI-HMM |
| 2766456 | 2779237 | 12781 | SIGI-HMM |
| 3892502 | 3897964 | 5462 | SIGI-HMM |
| 3907306 | 3913280 | 5974 | SIGI-HMM |
| 4404522 | 4409716 | 5194 | SIGI-HMM |
| 507821 | 517135 | 9314 | IslandPath-DIMOB |
| 628016 | 659365 | 31349 | IslandPath-DIMOB |
| 664467 | 671289 | 6822 | IslandPath-DIMOB |
| 678281 | 690692 | 12411 | IslandPath-DIMOB |
| 697335 | 724924 | 27589 | IslandPath-DIMOB |
| 739692 | 753026 | 13334 | IslandPath-DIMOB |
| 1767658 | 1773351 | 5693 | IslandPath-DIMOB |
| 2762572 | 2783419 | 20847 | IslandPath-DIMOB |
| 3263421 | 3273176 | 9755 | IslandPath-DIMOB |
| 3890826 | 3914156 | 23330 | IslandPath-DIMOB |
| 4387588 | 4401246 | 13658 | IslandPath-DIMOB |
| 4531507 | 4535124 | 3617 | IslandPath-DIMOB |
