# Supplementary material for: The Genome Sequence of Polymorphum gilvum SL003B-26A1T Reveals Its Genetic Basis for Crude Oil Degradation and Adaptation to the Saline Soil
Source: PLoS One. 2012 Feb 16;7(2):e31261. doi: 10.1371/journal.pone.0031261 (PMC3281065; doi:10.1371/journal.pone.0031261)
Supplement: Table S4 — Genes in predicted GIs by SIGI-HMM program. (DOC) [file pone.0031261.s006.doc]

## Table S4 Genes in predicted GIs by SIGI-HMM program

| **Locus_Tag （SL003B_*）** | **Strat** | **End** | **Production** | **Max match (nr)** | **Tax** | **Identities (%)** | **COG** |
| --- | --- | --- | --- | --- | --- | --- | --- |
| 0318 | 314738 | 317191 | SMC domain protein | YP_002975407.1 | *Azorhizobium caulinodans ORS 571* | 38.79 |  |
| 0319 | 318177 | 317356 | hypothetical protein | YP_001752710.1 | *Methylobacterium radiotolerans* JCM 2831 | 60 |  |
| 0320 | 318530 | 318207 | Similar to putative DNA-binding protein | YP_001260665.1 | Sphingomonas wittichii RW1 | 84.91 | COG2944 |
| 0321 | 318897 | 318508 | hypothetical protein | YP_001991178.1 | *Rhodopseudomonas palustris TIE-1* | 58.33 | COG4737 |
| 0480 | 491075 | 491341 | hypothetical protein | no match |  |  |  |
| 0481 | 491095 | 489137 | Peptidase, M23/M37 family protein | ZP_01545916.1 | *Stappia aggregata IAM 12614* | 59.32 | COG0739 |
| 0482 | 498381 | 498151 | hypothetical protein | no match |  |  |  |
| 0483 | 499364 | 500089 | Replication initiator and transcription repressor protein | AAX19278.1 | *Sinorhizobium meliloti* | 44.49 | COG5534 |
| 0484 | 500375 | 500545 | Probable insertion sequence transposase protein | ZP_03502746.1 | *Rhizobium etli Kim 5* | 83.93 |  |
| 0485 | 500683 | 501105 | Transposase and inactivated derivatives-like protein | CAB43594.1 | *Sinorhizobium meliloti* | 94.29 | COG3335 |
| 0486 | 502311 | 502090 | hypothetical protein | no match |  |  |  |
| 0487 | 504426 | 502402 | Plasmid transfer factor, traG | YP_757572.1 | *Maricaulis maris MCS10* | 30.52 | COG3505 |
| 0488 | 504960 | 505904 | Peptidase M, neutral zinc metallopeptidases, zinc-binding site | ZP_06351388.1 | *Rhodomicrobium vannielii ATCC 17100* | 52.58 | COG4227 |
| 0489 | 507821 | 506184 | Flagellar hook-basal body protein | YP_001917571.1 | *Natranaerobius thermophilus JW/NM-WN-LF* | 26.02 | COG1749 |
| 0516 | 537838 | 535070 | Prophage LambdaMc01, helicase, SNF2 family | YP_115086.1 | *Methylococcus capsulatus str. Bath* | 77.29 | COG0553 |
| 0517 | 541541 | 538344 | hypothetical protein | YP_994910.1 | *Verminephrobacter eiseniae EF01-2* | 69.48 | COG1743 |
| 0518 | 542544 | 541555 | hypothetical protein | YP_115089.1 | *Methylococcus capsulatus str. Bath* | 65.35 |  |
| 0519 | 544844 | 542544 | hypothetical protein | ZP_03544991.1 | *Comamonas testosteroni KF-1* | 70.83 |  |
| 0520 | 546179 | 545307 | COG2378: Predicted transcriptional regulator | ZP_01037725.1 | *Roseovarius sp. 217* | 58.89 | COG2378 |
| 0521 | 547291 | 547070 | hypothetical protein | ADO00837.1 | *Geobacter bemidjiensis Bem* | 42.86 |  |
| 0522 | 548525 | 547314 | Integrase family protein | YP_124435.1 | *Legionella pneumophila str. Paris* | 43.85 | COG0582 |
| 0523 | 549164 | 550243 | Transcriptional regulator, AraC family | YP_001892522.1 | *Ralstonia pickettii 12J* | 38.76 | COG2207 |
| 0524 | 550732 | 552204 | Serine protease, subtilase family | ZP_05813083.1 | *Mesorhizobium opportunistum WSM2075* | 30.95 |  |
| 0525 | 553091 | 552231 | Integrase catalytic region | YP_001415477.1 | *Xanthobacter autotrophicus Py2* | 75.79 | COG2801 |
| 0526 | 553360 | 553088 | transposase IS3/IS911 family protein | YP_001542277.1 | *Dinoroseobacter shibae DFL 12* | 70 | COG2963 |
| 0527 | 553426 | 553665 | Transcriptional regulator protein | YP_002540947.1 | *Agrobacterium radiobacter K84* | 84.51 |  |
| 0604 | 646003 | 644585 | Hypothetical conserved protein | YP_002433916.1 | *Desulfatibacillum alkenivorans AK-01* | 53.62 |  |
| 0605 | 647717 | 645996 | SMC domain protein | YP_002433915.1 | *Desulfatibacillum alkenivorans AK-01* | 58.32 |  |
| 0606 | 648897 | 649481 | Integrase family protein | YP_001923055.1 | *Methylobacterium populi BJ001* | 53.16 | COG4974 |
| 0617 | 659055 | 659276 | hypothetical protein | no match |  |  |  |
| 0618 | 660543 | 659365 | Acetate kinase protein | YP_002540634.1 | *Agrobacterium radiobacter K84* | 63.14 | COG0282 |
| 0619 | 661946 | 660540 | Phosphate acetyl/butyryltransferase family protein | YP_002287866.1 | *Oligotropha carboxidovorans OM5* | 70.43 | COG0280;COG2030 |
| 0620 | 663735 | 661954 | Poly-beta-hydroxybutyrate polymerase domain protein | YP_918859.1 | *Paracoccus denitrificans PD1222* | 67.42 | COG3243 |
| 0621 | 664322 | 663732 | Transcriptional regulator, TetR family | YP_783567.1 | *Rhodopseudomonas palustris BisA53* | 36.46 |  |
| 0622 | 664467 | 664811 | hypothetical protein | no match |  |  |  |
| 0623 | 665428 | 665171 | hypothetical protein | no match |  |  |  |
| 0624 | 665715 | 665425 | hypothetical protein | no match |  |  |  |
| 0625 | 666374 | 666625 | hypothetical protein | no match |  |  |  |
| 0626 | 667494 | 667832 | Hypothetical conserved protein | ZP_01048648.1 | *Nitrobacter sp. Nb-311A* | 54 | COG5489 |
| 0630 | 670085 | 670366 | Probable insertion sequence transposase protein | CAD31253.1 | *Mesorhizobium loti* | 96.59 | COG2801 |
| 0631 | 670360 | 671289 | Integrase catalytic region | CAD31272.1 | *Mesorhizobium loti* | 87.7 | COG2801 |
| 0632 | 671664 | 671305 | hypothetical protein | no match |  |  |  |
| 0633 | 672371 | 671685 | hypothetical protein | no match |  |  |  |
| 0634 | 673662 | 674105 | hypothetical protein | no match |  |  |  |
| 0637 | 678281 | 678054 | Putative uncharacterized protein | YP_003754501.1 | *Hyphomicrobium denitrificans ATCC 51888* | 54.39 |  |
| 0638 | 678837 | 678283 | Plasmid partition protein ParA-like protein | YP_001919383.1 | *Escherichia coli 53638* | 47.5 |  |
| 0639 | 679113 | 679880 | Transcriptional regulator, LuxR family | YP_428476.1 | *Rhodospirillum rubrum ATCC 11170* | 30.73 | COG2771 |
| 0640 | 680418 | 679813 | IS20 family transposase IstB-like protein | YP_003329379.1 | *Sinorhizobium meliloti* | 96.52 | COG1484 |
| 0641 | 682075 | 680426 | IS20 family transposase IstA-like protein (Fragment) | NP_355800.1 | *Agrobacterium tumefaciens str. C58* | 86.6 | COG4584 |
| 0642 | 682123 | 682332 | Transcriptional regulator, LuxR family | YP_528967.1 | *Saccharophagus degradans 2-40* | 52.27 |  |
| 0643 | 682438 | 683049 | Conjugation factor synthetase; TraI | ZP_06356988.1 | *Rhodopseudomonas palustris DX-1* | 42.7 | COG3916 |
| 0644 | 683930 | 683280 | Integrase family protein | YP_509478.1 | *Jannaschia sp. CCS1* | 50.75 | COG4974 |
| 0645 | 684998 | 685570 | Invertase recombinase-like protein | YP_422672.1 | *Magnetospirillum magneticum AMB-1* | 62.29 | COG1961 |
| 0646 | 686977 | 686033 | Putative protease HtpX family protein | ZP_05787241.1 | *Silicibacter lacuscaerulensis ITI-1157* | 54.37 | COG0501 |
| 0647 | 688105 | 686987 | Serine protease Do-like DegP (Trypsin-like protease with PDZ domain) | ZP_06897759.1 | *Roseomonas cervicalis ATCC 49957* | 61.52 | COG0265 |
| 0648 | 688593 | 688102 | Putative uncharacterized protein | YP_001372989.1 | *Ochrobactrum anthropi ATCC 49188* | 74.85 |  |
| 0649 | 689199 | 688708 | Heat shock protein Hsp20 | YP_001372990.1 | *Ochrobactrum anthropi ATCC 49188* | 95.09 | COG0071 |
| 0650 | 689618 | 689220 | Heat shock protein Hsp20 | YP_002967063.1 | *Methylobacterium extorquens AM1* | 96.21 | COG0071 |
| 0651 | 690058 | 689633 | Heat shock protein Hsp20 | |YP_001372992.1 | *Ochrobactrum anthropi ATCC 49188* | 96.85 | COG0071 |
| 0655 | 695587 | 696726 | Putative RNA polymerase, sigma 28 subunit, FliA/WhiG family | YP_002967068.1 | *Methylobacterium extorquens* AM1 | 69.97 |  |
| 0656 | 696726 | 697301 | ETC complex I subunit conserved region | YP_002967072.1 | *Methylobacterium extorquens* AM1 | 74.07 |  |
| 0657 | 697335 | 698087 | Rhomboid family protein | YP_002822314.1 | *Rhizobium* sp. NGR234 | 62.85 | COG0705 |
| 0658 | 698078 | 698920 | Periplasmic serine protease | YP_001243533.1 | *Bradyrhizobium* sp. BTAi1 | 78.18 | COG0616 |
| 0659 | 699180 | 699395 | Thioredoxin | YP_001373007.1 | *Ochrobactrum anthropi* ATCC 49188 | 87.32 |  |
| 0660 | 699444 | 700622 | Sodium/hydrogen exchanger | YP_001373008.1 | *Ochrobactrum anthropi* ATCC 49188 | 80.69 | COG0475 |
| 0661 | 701085 | 700864 | hypothetical protein | ADO00837.1 | *Geobacter bemidjiensis* Bem | 44.64 |  |
| 0667 | 708233 | 709432 | AAA ATPase, central region | YP_673081.1 | *Mesorhizobium sp. BNC1* | 61.83 | COG0464 |
| 0668 | 709436 | 711970 | hypothetical protein | YP_673080.1 | *Mesorhizobium sp. BNC1* | 58.16 | COG1404 |
| 0669 | 712721 | 712179 | hypothetical protein | no match |  |  |  |
| 0673 | 716282 | 719614 | Soluble lytic murein transglycosylase and regulatory protein | ZP_01551974.1 | *Methylophilales bacterium HTCC2181* | 30.52 |  |
| 0674 | 719708 | 720016 | Putative transposase related protein | ZP_07602127.1 | *Sinorhizobium meliloti AK83* | 93.14 | COG3293 |
| 0675 | 720034 | 720420 | Transposase IS4 family protein | YP_766066.1 | *Rhizobium leguminosarum bv. viciae 3841* | 89.06 | COG3293 |
| 0676 | 721048 | 721440 | Putative transposon | YP_973190.1 | *Polaromonas naphthalenivorans CJ2* | 55.17 |  |
| 0707 | 746435 | 745551 | Putative uncharacterized protein | YP_002945935.1 | *Variovorax paradoxus* S110 | 45.17 |  |
| 0708 | 746697 | 746455 | Putative uncharacterized protein | YP_001260703.1 | *Sphingomonas wittichii* RW1 | 80.26 |  |
| 0709 | 746799 | 747677 | Transcriptional regulator | YP_001237385.1 | *Bradyrhizobium* sp. BTAi1 | 60.56 | COG2378 |
| 0710 | 747733 | 749271 | Type I restriction modification system M subunit (Site-specific DNA-methyltransferase subunit) | ZP_05341231.1 | *Thalassiobium* sp. R2A62 | 73.83 | COG0286 |
| 0711 | 749268 | 750440 | Putative Restriction modification system, type I similar to hsdS | YP_001990515.1 | *Rhodopseudomonas palustris* TIE-1 | 43.14 | COG0732 |
| 0712 | 751754 | 750675 | Transposase (Class II) | YP_003189307.1 | *Acetobacter pasteurianus* IFO 3283-01 | 68.75 | COG3039 |
| 0713 | 752157 | 753026 | Putative uncharacterized protein | YP_001790348.1 | *Leptothrix cholodnii* SP-6 | 52.94 |  |
| 1006 | 1079267 | 1080844 | integral membrane sensor signal transduction histidine kinase | ZP_05783835.1 | *Citreicella sp. SE45* | 29.53 | COG0642 |
| 1007 | 1080822 | 1081505 | two component transcriptional regulator, winged helix family | YP_003692992.1 | *Starkeya novella DSM 506* | 47.11 | COG0745 |
| 1008 | 1081589 | 1082164 | hypothetical protein | ZP_05783703.1 | *Citreicella sp. SE45* | 36.17 |  |
| 1009 | 1082161 | 1082511 | putative exported protein | ZP_01880247.1 | *Roseovarius sp. TM1035* | 57.66 |  |
| 1010 | 1083438 | 1082617 | Secreted protein-like protein | ZP_02143299.1 | *Roseobacter litoralis Och 149* | 58.78 | COG5501 |
| 1135 | 1221849 | 1222190 | Integrase, catalytic region | YP_001045062.1 | *Rhodobacter sphaeroides ATCC 17029* | 81.08 |  |
| 1136 | 1222333 | 1223913 | Filamentation induced by cAMP protein Fic | ZP_02188240.1 | *alpha proteobacterium BAL199* | 71.46 | COG3177 |
| 1137 | 1225890 | 1224709 | Predicted tRNA(5-methylaminomethyl-2-thiouridylate) methyltransferase, contains the PP-loop ATPase domain | ZP_07659473.1 | *Roseibium sp. TrichSKD4* | 74.73 | COG0482 |
| 1138 | 1227436 | 1226240 | hypothetical protein | no match |  |  |  |
| 1416 | 1526662 | 1527558 | Putative taurine dioxygenase protein | YP_001240769.1 | *Bradyrhizobium sp. BTAi1* | 64.78 | COG2175 |
| 1417 | 1527591 | 1528943 | Coenzyme F420-dependent N5 N10-methylene tetrahydromethanopterin reductase and related flavin-dependent oxidoreductase-like protein | YP_918881.1 | *Paracoccus denitrificans PD1222* | 81.11 | COG2141 |
| 1418 | 1528957 | 1529790 | Probable aliphatic sulphonate ABC transporter, permease protein | YP_918882.1 | *Paracoccus denitrificans PD1222* | 62.75 | COG0600 |
| 1419 | 1529825 | 1530805 | Aliphatic sulfonate ABC transporter substrate-binding protein | YP_002979383.1 | *Rhizobium leguminosarum bv. trifolii WSM1325* | 73.93 | COG0715 |
| 1420 | 1530817 | 1531587 | ABC transporter related | YP_001312954.1 | *Sinorhizobium medicae WSM419* | 72.27 | COG1116 |
| 2589 | 2767286 | 2766456 | Integrase catalytic region | YP_001415477.1 | *Xanthobacter autotrophicus* Py2 | 79.66 | COG2801 |
| 2590 | 2767555 | 2767283 | ISEhe3 transposase A | YP_001542277.1 | *Dinoroseobacter shibae* DFL 12 | 70 | COG2963 |
| 2591 | 2767692 | 2767988 | Regulatory protein, LacI family | ZP_01034118.1 | *Roseovarius* sp. 217 | 78.49 |  |
| 2592 | 2768478 | 2768029 | DNA mismatch endonuclease Vsr | YP_001347881.1 | *Pseudomonas aeruginosa* PA7 | 54.74 | COG3727 |
| 2593 | 2773974 | 2768488 | hypothetical protein | YP_001768430.1 | *Methylobacterium* sp. 4-46 | 55.84 |  |
| 2594 | 2775491 | 2773971 | hypothetical protein | YP_001768431.1 | *Methylobacterium* sp. 4-46 | 55.44 |  |
| 2595 | 2777596 | 2775488 | hypothetical protein | YP_001768432.1 | *Methylobacterium* sp. 4-46 | 56.2 |  |
| 2596 | 2779042 | 2777600 | Modification methylase DdeI | NP_387254.1 | *Sinorhizobium meliloti* 1021 | 65.5 | COG0270 |
| 2597 | 2779237 | 2779055 | Prevent-host-death family protein | YP_001169959.1 | *Rhodobacter sphaeroides* ATCC 17025 | 57.63 |  |
| 3592 | 3892502 | 3892735 | hypothetical protein | ZP_05113135.1 | *Labrenzia alexandrii* DFL-11 | 53.33 |  |
| 3593 | 3894693 | 3893281 | Piwi domain protein | NP_952414.1 | *Geobacter sulfurreducens* PCA | 54.12 |  |
| 3594 | 3896474 | 3894690 | hypothetical protein | NP_952413.1 | *Geobacter sulfurreducens PCA* | 41.99 |  |
| 3595 | 3897526 | 3896471 | Bacterial regulatory proteins, AsnC family | NP_842120.1 | *Nitrosomonas europaea* ATCC 19718 | 68.38 | COG3177 |
| 3596 | 3897964 | 3897605 | hypothetical protein | YP_002362277.1 | *Methylocella silvestris* BL2 | 51.38 |  |
| 3605 | 3907530 | 3907306 | hypothetical protein | ZP_00964814.1 | *Sulfitobacter sp. NAS-14.1* | 66.67 |  |
| 3606 | 3908225 | 3908962 | hypothetical protein | no match |  |  |  |
| 3607 | 3911970 | 3909088 | hypothetical protein | XP_002506687.1 | *Micromonas sp. RCC299* | 26.58 |  |
| 3608 | 3912026 | 3912535 | hypothetical protein | no match |  |  |  |
| 3609 | 3912996 | 3913280 | hypothetical protein | no match |  |  |  |
| 4099 | 4404522 | 4406057 | Dehydrogenase PhnF | YP_913992.1 | *Paracoccus denitrificans* PD1222 | 67.08 | COG1012 |
| 4100 | 4406239 | 4407483 | Putative cytochrome p450-like enzyme | ZP_01744008.1 | *Sagittula stellata* E-37 | 36.52 | COG2124 |
| 4101 | 4407598 | 4408455 | 2-hydroxyhepta-2,4-diene-1,7-dioate isomerase | YP_001630034.1 | *Bordetella petrii* DSM 12804 | 60.56 | COG0179 |
| 4102 | 4409032 | 4408508 | MarR family transcription regulator protein | YP_003777372.1 | *Herbaspirillum seropedicae* SmR1 | 31.25 | COG1846 |
| 4103 | 4409393 | 4409716 | Probable ferredoxin, 2fe-2s fdii electron transport iron-sulfur protein | BAC98954.1 | *Xanthobacter polyaromaticivorans* | 42.99 | COG0633 |
