# Supplementary material for: The Genome Sequence of Polymorphum gilvum SL003B-26A1T Reveals Its Genetic Basis for Crude Oil Degradation and Adaptation to the Saline Soil
Source: PLoS One. 2012 Feb 16;7(2):e31261. doi: 10.1371/journal.pone.0031261 (PMC3281065; doi:10.1371/journal.pone.0031261)
Supplement: Table S5 — Insertion sequences predicted in SL003B-26A1T. (DOC) [file pone.0031261.s007.doc]

## Table S5 Insertion sequences predicted in SL003B-26A1T

| Elements | Locus_Tag (SL003B_*) |
| --- | --- |
| Transposases |  |
| IS3 | 0495,0526,0630, 1661, 1662, 2275, 2586, 2590 |
| IS5 | 0492, 0494, 0674, 0675, 0712, 0800, 0801, 1718, 3035, 3138 |
| IS21 | 0263, 0264, 0265, 0640, 0641, 0714, 0715, 1057, 2439, 2440, 2837, 2838, 3180, 3181, 4073, 4075, 4076, p0022, p0055, p0021  p0054 |
| IS66 | 4112, 4113, 4114, p0027, p0026, p0024 |
| IS630 | 0390, 0391, 0484, 0485, 0684, 0685, 0698, 0699 |
| Others | 0686, 3770, 4074, 1948, 4085, 0706, 4136, 0262, 4086, 4008 |
| Integrases | 0322, 0522, 0525, 0606, 0611, 0631, 0644, 0662, 0670, 0672, 0689, 0704, 0813, 1135, 1704, 2274, 2276, 2438, 2587, 2588, 2589, 2640, 3799, 3848, 4009, 4077, 4221 |
