# Supplementary material for: The Genome Sequence of Polymorphum gilvum SL003B-26A1T Reveals Its Genetic Basis for Crude Oil Degradation and Adaptation to the Saline Soil
Source: PLoS One. 2012 Feb 16;7(2):e31261. doi: 10.1371/journal.pone.0031261 (PMC3281065; doi:10.1371/journal.pone.0031261)
Supplement: Table S6 — Transcription (COG category K). (DOC) [file pone.0031261.s008.doc]

## Table S6 Transcription (COG category K)

| **Protein** | **COG** | **Number of proteins** | **Locus_Tag** |
| --- | --- | --- | --- |
| ATP-dependent DNA helicase RecG | COG1200 | 1 | 2325 |
| Cold-shock DNA-binding domain protein | COG1278 | 5 | 1226, 1984, 3547, 3721, 4222 |
| Condensin subunit ScpB | COG1386 | 1 | 2193 |
| CopG-like protein | COG3609 | 3 | 1371, 3758, 4129 |
| Cytochrome c oxidase, subunit I | COG2183 | 1 | 0966 |
| DEAD/DEAH box helicase domain protein | COG0513 | 3 | 2355, 3230, 3350 |
| DNA-directed RNA polymerase subunit alpha | COG0202 | 1 | 1763 |
| DNA-directed RNA polymerase subunit beta | COG0085 | 1 | 1730 |
| DNA-directed RNA polymerase subunit beta | COG0086 | 1 | 1731 |
| DNA-directed RNA polymerase subunit omega | COG1758 | 1 | 1900 |
| GTP pyrophosphokinase | COG0317 | 1 | 1899 |
| Heat-inducible transcription repressor hrcA | COG1420 | 1 | 0022 |
| Helicase domain protein | COG0553 | 3 | 0516, 1717, 3625 |
| HxlR-like helix-turn-helix protein | COG1733 | 1 | 2965 |
| Hypothetical protein | COG2740 | 1 | 4303 |
| Hypothetical protein | COG3423 | 2 | 1656, 4013 |
| Methyltransferase type 11 | COG4646 | 1 | p0009 |
| NAD-dependent deacetylase 1 | COG0846 | 1 | 1983 |
| Nitrous-oxide reductase transcriptional activator NosR | COG3901 | 2 | 0565, 0577 |
| ParB-like nuclease domain protein | COG1475 | 9 | 0080, 0314, 0500, 0616, 0677, 1658, 4011, p0011, p0047 |
| Peptidase S24-like domain protein | COG2932 | 1 | 3261 |
| Phage integrase family site-specific recombinase | COG1733 | 1 | 0611 |
| Phage transcriptional regulator, AlpA | COG3311 | 1 | 0300 |
| Putative ery operon repressor transcription regulator protein | COG2390 | 1 | 3639 |
| Putative kinase/transcriptional regulator, actin-like ATPase domain (NagC/XylR (ROK) familiy) | COG1940 | 2 | 0747, 2652 |
| Putative transcription regulator protein | COG1813 | 1 | 2797 |
| Putative transcription regulator protein | COG2345 | 1 | 1300 |
| Putative transcription regulator protein | COG2378 | 3 | 0520, 0709, 3940 |
| Putative transcription regulator protein | COG3905 | 1 | 0873 |
| Putative transcription regulator protein | COG5631 | 1 | 1327 |
| Putative transcriptional activator | COG0819 | 1 | 2357 |
| Putative transcriptional regulator protein | COG1695 | 1 | 2726 |
| Putative transcriptional regulator, consists of a Zn-ribbon and ATP-cone domains | COG1327 | 1 | 1917 |
| Putative transcriptional regulator, CopG family | COG0864 | 1 | 1447 |
| Response regulator of hydrogenase 3 activity (Sensor HydH) | COG3829 | 1 | 3819 |
| Response regulators consisting of a CheY-like receiver domain | COG0745 | 14 | 0156, 0539, 0829, 0954, 1007, 1203, 2580, 2901, 2913, 3001, 3275, 3847, 4028, 4206 |
| Response regulators consisting of a CheY-like receiver domain | COG2197 | 7 | 0821, 1003, 2123, 2983, 3223, 3941, p0002 |
| Response regulators consisting of a CheY-like receiver domain | COG3437 | 5 | 0235, 0411, 2367, 3205, 3537 |
| Response regulators consisting of a CheY-like receiver domain | COG4567 | 1 | 0018 |
| RNA polymerase sigma factor | COG0568 | 1 | 0395 |
| RNA polymerase sigma factor | COG1595 | 3 | 0176, 1060, 3966 |
| RNA polymerase sigma factor, sigma-70 family | COG1595 | 1 | 1422 |
| RNA polymerase, sigma 28 subunit | COG1595 | 1 | 0655 |
| RNA polymerase, sigma 32 subunit, RpoH | COG0568 | 1 | 0938 |
| RNA polymerase, sigma 54 subunit, RpoN/SigL | COG1508 | 1 | 3933 |
| RNA polymerase, sigma 70 subunit, RpoD | COG0568 | 1 | 1266 |
| RNAse III | COG0571 | 1 | 1894 |
| RNAse R | COG0557 | 1 | 1845 |
| ROS/MUCR transcriptional regulator protein | COG4957 | 1 | 2954 |
| SOS-response transcriptional repressors (RecA-mediated autopeptidases) | COG1974 | 1 | 2110 |
| Transcription antitermination protein, NusB | COG0781 | 1 | 1921 |
| Transcription antitermination protein, NusG1 | COG0250 | 1 | 1725 |
| Transcription elongation factor GreA | COG0782 | 1 | 1258 |
| Transcription elongation factor NusA | COG0195 | 1 | 4305 |
| Transcription termination factor Rho protein | COG1158 | 1 | 0074 |
| Transcriptional regulator, ArsR family | COG3327 | 1 | 2144 |
| Transcriptional regulator, BadM/Rrf2 family | COG1959 | 5 | 0324, 1406, 2635, 3050, 3574 |
| Transcriptional regulator, CarD family | COG1329 | 1 | 0396 |
| Transcriptional regulator, DeoR family | COG1349 | 3 | 0741, 1063, 3108 |
| Transcriptional regulator, Fis family | COG3284 | 1 | 3715 |
| Transcriptional regulator, LacI family | COG1609 | 3 | 0806, 1032, 3191 |
| Transcriptional regulator, LuxR family | COG2771 | 5 | 0401, 0514, 0642, 0701, 3298 |
| Transcriptional regulator, RpiR family | COG1737 | 3 | 1102, 3553, 4142 |
| Transcriptional regulator, XRE family | COG1395 | 1 | 3430 |
| Transcriptional regulator, XRE family | COG1396 | 2 | 4250, 4309 |
| Transcriptional regulator, XRE family | COG1476 | 2 | 0968, 1712 |
| Transcriptional regulator, XRE family | COG3620 | 3 | 0861, 1260, 3004 |
| Transcriptional regulator, XRE family | COG2944 | 1 | 320 |
| Transcriptional regulators, AraC family | COG2207 | 11 | 0532, 0726, 1261, 1505, 2161, 2491, 2723, 3128, 3246, 3252, 3953 |
| Transcriptional regulators, AraC family | COG4977 | 2 | 0523, 2770 |
| Transcriptional regulators, ArsR family | COG0640 | 6 | 0941, 1328, 2691, 2732, 2741, 3780 |
| Transcriptional regulators, AsnC family | COG1522 | 11 | 0570, 0571, 0572, 0757, 1257, 1411, 1433, 1477, 1593, 2823, 3702 |
| Transcriptional regulators, GntR family | COG1167 | 2 | 3251, 3353 |
| Transcriptional regulators, GntR family | COG1802 | 10 | 1958, 2492, 2538, 2649, 2862, 3070, 3187, 3524, 3585, 3588 |
| Transcriptional regulators, GntR family | COG2186 | 5 | 1126, 2449, 2523, 3197, 3481 |
| Transcriptional regulators, GntR family | COG2188 | 2 | 0429, 3839 |
| Transcriptional regulators, IclR family | COG1414 | 6 | 1054, 2469, 2480, 3118, 3144, p0028 |
| Transcriptional regulators, LysR family | COG0583 | 32 | 0275, 0552, 0717, 0720, 0815, 0993, 1103, 1107, 1121, 1188, 1240, 1254, 1301, 1862, 2126, 2180, 2246, 2505, 2528, 2713, 2851, 2877, 3139, 3151, 3163, 3179, 3478, 3487, 3639, 3846, 4016, 4270 |
| Transcriptional regulators, MarR family | COG1846 | 21 | 0221, 0608, 0652, 0798, 1204, 1236, 1292, 1807, 1857, 1913, 1981, 2431, 2639, 2674, 2745, 2868, 3407, 3655, 3802, 4102, p0068 |
| Transcriptional regulators, MerR family | COG0789 | 6 | 1262, 1614, 1802, 1931, 3240, 4228 |
| Transcriptional regulators, TetR family | COG1309 | 19 | 0183, 0268, 0386, 0621, 1071, 1081, 1398, 1472, 2143, 2152, 2285, 2288, 2289, 2497, 2619, 3204, 3494, 3552, 3569 |
| Transcription-repair coupling factor | COG1197 | 1 | 2323 |
| Two-component response regulator | COG1595 | 1 | 1058 |
| UPF0301 protein SADFL11_5049 | COG1678 | 1 | 3389 |
| Urease accessory protein UreG | COG0378 | 1 | 0098 |
| **Total** |  | **271** |  |
