# Supplementary material for: The Genome Sequence of Polymorphum gilvum SL003B-26A1T Reveals Its Genetic Basis for Crude Oil Degradation and Adaptation to the Saline Soil
Source: PLoS One. 2012 Feb 16;7(2):e31261. doi: 10.1371/journal.pone.0031261 (PMC3281065; doi:10.1371/journal.pone.0031261)
Supplement: Table S7 — Transporters. (DOC) [file pone.0031261.s009.doc]

## Table S7 Transporters

| **TC-DB Number** | **Transporter Classification Family Name** | **Number of Proteins** | **Locus_Tag** |
| --- | --- | --- | --- |
| **1.A.** | **α-Channels** | | |
| TC:1.A.1 | Voltage-gated Ion Channel (VIC) Superfamily | 3 | 1562, 3265, 4168 |
| TC:1.A.11 | The Ammonia Transporter Channel (Amt) Family | 2 | 0244, 3434 |
| TC:1.A.22 | The Large Conductance Mechanosensitive Ion Channel (MscL) Family | 1 | 3221 |
| TC:1.A.23 | The Small Conductance Mechanosensitive Ion Channel (MscS) Family | 1 | 0959 |
| TC:1.A.30 | The H^+^- or Na^+^-translocating Bacterial Flagellar Motor/ExbBD Outer Membrane Transport Energizer (Mot/Exb) Superfamily | 13 | 841, 842, 898, 0921, 0922, 2168, 2169, 2170, 2262, 2263, 2264, 3853, 3879 |
| TC:1.A.33 | The Cation Channel-forming Heat Shock Protein-70 (Hsp70) Family | 2 | 3893, 4327 |
| TC:1.A.35 | The CorA Metal Ion Transporter (MIT) Family | 1 | 3288 |
| TC:1.A.62 | The Homotrimeric Cation Channel (TRIC) Family | 1 | 4193 |
| **1.B** | **β-Barrel porins** | | |
| TC:1.B.14 | The Outer Membrane Receptor (OMR) Family | 2 | 2162, 2266 |
| TC:1.B.17 | The Outer Membrane Factor (OMF) Family | 1 | 2038 |
| TC:1.B.18 | The Outer Membrane Auxiliary (OMA) Protein Family | 4 | 1935, 2541, 2792, 3022 |
| TC:1.B.20 | The Two-Partner Secretion (TPS) Family | 1 | 1086 |
| TC:1.B.22 | The Outer Bacterial Membrane Secretin (Secretin) Family | 1 | 3663 |
| TC:1.B.33 | The Outer Membrane Protein Insertion Porin (Bam Complex) (OmpIP) Family | 4 | 1355, 1925, 2101, 2795 |
| TC:1.B.39 | The Bacterial Porin, OmpW (OmpW) Family | 2 | 4078, 4109 |
| TC:1.B.42 | The Outer Membrane Lipopolysaccharide Export Porin (LPS-EP) Family | 5 | 2393, 2394, 2395, 3935, 3936 |
| TC:1.B.46 | The Outer Membrane LolAB Lipoprotein Insertion Apparatus (LolAB) Family | 1 | 0141 |
| TC:1.B.54 | The Intimin/Invasin (Int/Inv) or Autotransporter-3 (AT-3) Family | 1 | 0673 |
| TC:1.B.9 | The FadL Outer Membrane Protein (FadL) Family | 2 | 1482, 1483 |
|  |  | | |
| TC:1.E.14 | The LrgA Holin (LrgA Holin) Family | 1 | 1601 |
| **2.A** | **Porters (uniporters, symporters, antiporters)** | | |
| TC:2.A.1 | The Major Facilitator Superfamily (MFS) | 16 | 0222, 0610, 0785, 1206, 1231, 1490, 1504, 1516, 1923, 2811, 2850, 2967, 3300, 3336, 3681, 4177 |
| TC:2.A.16 | The Telurite-resistance/Dicarboxylate Transporter (TDT) Family | 1 | 2907 |
| TC:2.A.19 | The Ca^2+^:Cation Antiporter (CaCA) Family | 2 | 1216, 1454 |
| TC:2.A.20 | The Inorganic Phosphate Transporter (PiT) Family | 1 | 1945 |
| TC:2.A.21 | The Solute:Sodium Symporter (SSS) Family | 1 | 2507 |
| TC:2.A.3 | The Amino Acid-Polyamine-Organocation (APC) Family | 1 | 3432 |
| TC:2.A.36 | The Monovalent Cation:Proton Antiporter-1 (CPA1) Family | 1 | 3008 |
| TC:2.A.37 | The Monovalent Cation:Proton Antiporter-2 (CPA2) Family | 3 | 0172, 0660, 3400 |
| TC:2.A.38 | The K^+^ Transporter (Trk) Family | 2 | 0097, 1618 |
| TC:2.A.4 | The Cation Diffusion Facilitator (CDF) Family | 3 | 0246, 3779, 4227 |
| TC:2.A.47 | The Divalent Anion:Na+ Symporter (DASS) Family | 4 | 0380, 0807, 1185, 1905 |
| TC:2.A.49 | The Chloride Carrier/Channel (ClC) Family | 1 | 1364 |
| TC:2.A.5 | The Zinc (Zn^2+^)-Iron (Fe^2+^) Permease (ZIP) Family | 1 | 0995 |
| TC:2.A.51 | The Chromate Ion Transporter (CHR) Family | 1 | 2569 |
| TC:2.A.52 | The Ni^2+^-Co^2+^ Transporter (NiCoT) Family | 1 | 0816 |
| TC:2.A.53 | The Sulfate Permease (SulP) Family | 3 | 3005, 3182, 4259 |
| TC:2.A.56 | The Tripartite ATP-independent Periplasmic Transporter (TRAP-T) Family | 77 | 0533, 0534, 0592, 0593, 0594, 0762, 0763, 0764, 0782, 0783, 0823, 0824, 0825, 0826, 0827, 1052, 1053, 1088, 1089, 1100, 1101, 1127, 1128, 1129, 1321, 1322, 1323, 1373, 1374, 1375, 1480, 2454, 2455, 2474, 2475, 2476, 2493, 2494, 2495, 2501, 2502, 2503, 2710, 2711, 2712, 2853, 2854, 2864, 2865, 2866, 3147, 3148, 3149, 3170, 3171, 3172, 3184, 3185, 3186, 3199, 3200, 3201, 3248, 3249, 3250, 3555, 3556, 3557, 3587, 3732, 3733, 3734, 4092, 4093, P0029, P0030, P0031 |
| TC:2.A.58 | The Phosphate:Na^+^ Symporter (PNaS) Family | 1 | 1337 |
| TC:2.A.59 | The Arsenical Resistance-3 (ACR3) Family | 1 | 0943 |
| TC:2.A.6 | The Resistance-Nodulation-Cell Division (RND) Superfamily | 9 | 0266, 1125, 1219, 2204, 2205, 2216, 2728, 3269, 3492 |
| TC:2.A.64 | The Twin Arginine Targeting (Tat) Family | 3 | 2195, 2196, 2197 |
| TC:2.A.66 | The Multidrug/Oligosaccharidyl-lipid/Polysaccharide (MOP) Flippase Superfamily | 7 | 0167, 0882, 3040, 3264, 3915, 4207, 4248 |
| TC:2.A.69 | The Auxin Efflux Carrier (AEC) Family | 3 | 0234, 1852, 2241 |
| TC:2.A.7 | The Drug/Metabolite Transporter (DMT) Superfamily | 14 | 0137, 0362, 0450, 0803, 0933, 1461, 1475, 2735, 2812, 3007, 3443, 3486, 3643, 4175 |
| TC:2.A.76 | The Resistance to Homoserine/Threonine (RhtB) Family | 5 | 1310, 2012, 2650, 3325, 4210 |
| TC:2.A.78 | The Branched Chain Amino Acid Exporter (LIV-E) Family | 3 | 1828, 1829, 3506 |
| TC:2.A.80 | The Tricarboxylate Transporter (TTT) Family | 5 | 0541, 1117, 1119, 2478, 2481 |
| TC:2.A.83 | The Na^+^-dependent Bicarbonate Transporter (SBT) Family | 1 | 0350 |
| TC:2.A.86 | The Autoinducer-2 Exporter (AI-2E) Family | 3 | 1274, 2382, 2962 |
| TC:2.A.88 | Vitamin Uptake Transporter (VUT or ECF) Family | 1 | 0599 |
| TC:2.A.89 | The Vacuolar Iron Transporter (VIT) Family | 2 | 1183, 2751 |
| TC:2.A.95 | The 6TMS Neutral Amino Acid Transporter (NAAT) Family | 1 | 2217 |
| TC:2.A.99 | The 6TMS Ni^2+^ uptake transporter (HupE-UreJ) Family | 1 | 2028 |
| **3.A** | **P-P-bond-hydrolysis-driven transporters** | | |
| TC:3.A.1 | The ATP-binding Cassette (ABC) Superfamily | 313 | 0039, 0115, 0130, 0131, 0251, 0252, 0413, 0414, 0415, 0416, 0417, 0423, 0424, 0442, 0548, 0549, 0550, 0551, 0561, 0562, 0563, 0596, 0597, 0657, 0730, 0731, 0732, 0733, 0734, 0737, 0738, 0739, 0740, 0748, 0749, 0750, 0770, 0771, 0772, 0773, 0792, 0793, 0832, 0833, 0834, 0835, 0836, 0891, 0936, 0960, 0961, 0962, 0963, 0964, 0967, 0970, 0971, 0974, 0975, 0976, 0978, 0979, 0980, 0981, 1000, 1001, 1018, 1023, 1027, 1028, 1029, 1030, 1040, 1041, 1042, 1043, 1044, 1074, 1075, 1076, 1077, 1079, 1111, 1112, 1113, 1167, 1170, 1275, 1276, 1277, 1293, 1394, 1407, 1408, 1410, 1418, 1420, 1442, 1443, 1445, 1446, 1469, 1470, 1494, 1502, 1503, 1597, 1637, 1866, 1867, 1868, 1869, 1870, 1873, 1874, 1875, 1876, 1878, 1879, 1880, 1881, 1963, 1966, 1967, 1968, 2004, 2006, 2007, 2008, 2024, 2089, 2090, 2140, 2142, 2156, 2157, 2158, 2159, 2160, 2164, 2165, 2166, 2174, 2176, 2177, 2178, 2194, 2236, 2251, 2252, 2253, 2254, 2268, 2269, 2270, 2294, 2295, 2296, 2305, 2306, 2336, 2375, 2389, 2422, 2423, 2424, 2425, 2428, 2442, 2443, 2444, 2445, 2447, 2457, 2458, 2459, 2460, 2485, 2518, 2519, 2520, 2521, 2522, 2529, 2530, 2531, 2534, 2546, 2547, 2571, 2572, 2573, 2642, 2643, 2644, 2645, 2681, 2705, 2706, 2779, 2780, 2781, 2818, 2819, 2820, 2834, 2835, 2836, 2849, 2875, 2882, 2883, 2884, 2885, 2887, 2888, 2889, 2890, 2993, 3069, 3084, 3085, 3087, 3092, 3093, 3111, 3112, 3113, 3114, 3123, 3124, 3125, 3126, 3127, 3130, 3131, 3132, 3133, 3134, 3150, 3158, 3159, 3192, 3193, 3195, 3267, 3277, 3278, 3279, 3280, 3514, 3515, 3519, 3520, 3521, 3522, 3541, 3542, 3543, 3544, 3545, 3566, 3567, 3568, 3573, 3576, 3577, 3578, 3579, 3580, 3672, 3689, 3690, 3691, 3692, 3705, 3805, 3806, 3807, 3808, 3809, 3813, 3814, 3815, 3816, 3817, 3834, 3835, 3836, 3837, 3838, 3842, 3843, 3844, 3845, 3866, 3934, 3947, 3948, 3949, 3950, 3951, 3969, 4025, 4138, 4139, 4178, 4179, 4180, 4181, 4285, 4288, 4290 |
| TC:3.A.10 | The H^+^-translocating Pyrophosphatase (H^+^-PPase) Family | 1 | 1924 |
| TC:3.A.12 | The Septal DNA Translocator (S-DNA-T) Family | 1 | 0142 |
| TC:3.A.15 | The Outer Membrane Protein Secreting Main Terminal Branch (MTB) Family | 1 | 3606 |
| TC:3.A.2 | The H^+^- or Na^+^-translocating F-type, V-type and A-type ATPase (F-ATPase) Superfamily | 12 | 0074, 0955, 3358, 3359, 3360, 3361, 3849, 4214, 4215, 4216, 4217, 4218 |
| TC:3.A.3 | The P-type ATPase (P-ATPase) Superfamily | 8 | 0769, 0999, 2630, 3096, 3206, 3795, 4015, 4019 |
| TC:3.A.5 | The General Secretory Pathway (Sec) Family | 7 | 0096, 0160, 0459, 1724, 1759, 2187, 2203 |
| TC:3.A.6 | The Type III (Virulence-related) Secretory Pathway (IIISP) Family | 20 | 1143, 1145, 1146, 1148, 1540, 1544, 1545, 1549, 1550, 1551, 1798, 2608, 3854, 3856, 3858, 3864, 3865, 3869, 3881, 3883 |
| TC:3.A.7 | The Type IV (Conjugal DNA-Protein Transfer or VirB) Secretory Pathway (IVSP) Family | 22 | 0277, 0278, 0279, 0280, 0283, 0284, 0285, 0290, 0487, 0513, 0601, 0636, 0665, 0695, 3604, P0034, P0036, P0037, P0038, P0041, P0042, P0043 |
| TC:3.A.8 | The Mitochondrial Protein Translocase (MPT) Family | 1 | 4253 |
| **3.D** | **Oxidoreduction-driven transporters** | | |
| TC:3.D.2 | The Proton-translocating Transhydrogenase (PTH) Family | 1 | 0589 |
| **4.A** | **Phosphotransfer-driven group translocators** | | |
| TC:4.A.6 | The PTS Mannose-Fructose-Sorbose (Man) Family | 1 | 4203 |
| **4.C** | **Acyl CoA ligase-coupled transporters** | | |
| TC:4.C.1 | The Proposed Fatty Acid Transporter (FAT) Family | 9 | 0537, 0887, 1460, 2235, 2870, 3075, 3285, 3550, 3811 |
| **5.A** | **Transmembrane 2-electron transfer carriers** | | |
| TC:5.A.1 | The Disulfide Bond Oxidoreductase D (DsbD) Family | 2 | 2331, 2693 |
| TC:5.A.2 | The Disulfide Bond Oxidoreductase B (DsbB) Family | 1 | 0433 |
| TC:5.A.3 | The Prokaryotic Molybdopterin-containing Oxidoreductase (PMO) Family | 5 | 1279, 2074, 2129, 2333, 2385 |
| TC:5.A.4 | The Prokaryotic Succinate Dehydrogenase (SDH) Family | 6 | 0184, 0185, 0187, 2525, 2537, 3827 |
| **5.B** | **Transmembrane 1-electron transfer carriers** | | |
| TC:5.B.1 | The Phagocyte (gp91^phox^) NADPH Oxidase Family | 3 | 0154, 2565, 4026 |
| **8.A** | **Auxiliary transport proteins** | | |
| TC:8.A.1 | The Membrane Fusion Protein (MFP) Family | 10 | 0267, 0609, 1124, 1218, 1471, 1501, 2304, 2727, 2848, 3493 |
| TC:8.A.21 | The Stomatin/Podocin/Band 7/Nephrosis.2/SPFH (Stomatin) Family | 4 | 0839, 1380, 1381, 3945 |
| TC:8.A.3 | The Cytoplasmic Membrane-Periplasmic Auxiliary-1 (MPA1) Protein with Cytoplasmic (C) Domain (MPA1-C or MPA1+C) Family | 3 | 1936, 3024, 3910 |
| TC:8.A.4 | The Cytoplasmic Membrane-Periplasmic Auxiliary-2 (MPA2) Family | 2 | 2544, 2545 |
| **9.A** | **Recognized transporters of unknown biochemical mechanism** | | |
| TC:9.A.18 | The Putative Peptide Uptake or Activated Fatty Acid Export Permease (PUP) Family | 1 | 1474 |
| TC:9.A.19 | The Mg^2+^ Transporter-E (MgtE) Family | 1 | 1800 |
| TC:9.A.29 | The Putative 4-Toluene Sulfonate Uptake Permease (TSUP) Family | 8 | 0366, 0888, 0915, 1050, 1320, 2832, 3238, 3507 |
| TC:9.A.4 | The YggT or Fanciful K^+^ Uptake-B (FkuB; YggT) Family | 1 | 0256 |
| TC:9.A.40 | The HlyC/CorC (HCC) Family | 4 | 0345, 0897, 1987, 4311 |
| TC:9.A.41 | The Capsular Polysaccharide Exporter (CPS-E) Family | 4 | 2540, 2789, 2790, 2791 |
| **9.B** | **Putative transport proteins** | | |
| TC:9.B.14 | The Putative Heme Handling Protein (HHP) Family | 2 | 0250, 2907 |
| TC:9.B.18 | The SecDF-associated Single Transmembrane Protein, YajC (YajC) Family | 1 | 2203 |
| TC:9.B.22 | The Leukotoxin Secretion Morphogenesis Protein C (MorC) Family | 1 | 2796 |
| TC:9.B.24 | The Testis-Enhanced Gene Transfer (TEGT) Family | 1 | 0440 |
| TC:9.B.27 | The DedA or YdjX-Z (DedA) Family | 1 | 4264 |
| TC:9.B.28 | The Putative Permease Duf318 (Duf318) Family | 3 | 2172, 2173, 2338 |
| TC:9.B.3 | The Putative Bacterial Murein Precursor Exporter (MPE) Family | 1 | 1346 |
| TC:9.B.30 | The Hly III (Hly III) Family | 1 | 3073 |
| TC:9.B.35 | The Putative Thyronine-Transporting Transthyretin (Transthyretin) Family | 1 | 2247 |
| TC:9.B.36 | The Acid Resistance Membrane Protein (HdeD) Family | 1 | 1567 |
| TC:9.B.43 | The YedZ (YedZ) Family | 3 | 0154, 2565, 4026 |
| TC:9.B.62 | The Copper Resistance (CopD) Family | 1 | 2566 |
| TC:9.B.67 | The Putative Inorganic Carbon (HCO3-) Transporter/O-antigen Polymerase (ICT/OAP) Family | 2 | 1932, 3026 |
| TC:9.B.7 | The Putative Sulfate Transporter (CysZ) Family | 1 | 0188 |
| TC:9.B.71 | The Camphor Resistance (CrcB) Family | 1 | 1773 |
| TC:9.B.78 | The Minor Capsid Protein, gp7 of Baccilus subtilis Phage SPP1 (gp7) Family | 2 | 1680, 3991 |
| TC:9.B.85 | The Outer Membrane Lipoprotein-A (OmlA) Family | 1 | 1925 |
| **Total** |  | 704 |  |
