# Supplementary material for: The Genome Sequence of Polymorphum gilvum SL003B-26A1T Reveals Its Genetic Basis for Crude Oil Degradation and Adaptation to the Saline Soil
Source: PLoS One. 2012 Feb 16;7(2):e31261. doi: 10.1371/journal.pone.0031261 (PMC3281065; doi:10.1371/journal.pone.0031261)
Supplement: Table S8 — Genes in biosurfactant synthesis. (DOC) [file pone.0031261.s010.doc]

## Table S8 Genes in biosurfactant synthesis

| **Encoded Protein** | **Start** | **End** | **Locus_Tag** | **Gene** | **COG** |
| --- | --- | --- | --- | --- | --- |
| Dehydrogenase with different specificities | 2561535 | 2562272 | 2404 |  | COG1028 |
| Short-chain dehydrogenase/reductase SDR | 952935 | 953726 | 0890 |  | COG1028 |
| phosphoglucomutase/phosphomannomutase, C-terminal domain family | 3979875 | 3981374 | 3676 |  | COG1109 |
| Thioesterase superfamily protein | 2450874 | 2451302 | 2316 |  | COG0824 |
| AMP-dependent synthetase and ligase | 381861 | 383498 | 0383 | acs | COG0365 |
| AMP-dependent synthetase and ligase | 1562376 | 1564049 | 1460 | lcfA | COG0318 |
| poly(R)-hydroxyalkanoic acid synthase, class I subfamily | 2846517 | 2848322 | 2662 |  | COG3243 |
| 3-oxoacyl-(acyl-carrier-protein) synthase 2 | 2559711 | 2560979 | 2402 |  | COG0304 |
| phosphoglucomutase/phosphomannomutase, C-terminal domain family | 3979875 | 3981374 | 3676 |  | COG1109 |
| Aspartokinase | 478963 | 480213 | 0473 |  | COG0527 |
| Fatty acid synthase transmembrane protein | 2982030 | 2989364 | 2786 |  | COG3321 |
| AMP-dependent synthetase and ligase | 3313018 | 3314532 | 3075 |  | COG0318 |
| Proline dehydrogenase family | 4005890 | 4009018 | 3701 |  | COG4230 |
| acetyl-coenzyme A synthetase | 176631 | 178271 | 0169 |  | COG0365 |
| AMP-dependent synthetase and ligase | 4126643 | 4128286 | 3811 |  | COG0318 |
| AMP-dependent synthetase and ligase | 1171195 | 1172976 | 1091 |  | COG0365 |
