# Supplementary material for: The Genome Sequence of Polymorphum gilvum SL003B-26A1T Reveals Its Genetic Basis for Crude Oil Degradation and Adaptation to the Saline Soil
Source: PLoS One. 2012 Feb 16;7(2):e31261. doi: 10.1371/journal.pone.0031261 (PMC3281065; doi:10.1371/journal.pone.0031261)
Supplement: Table S9 — Genes in hydrocarbon and aromatic compounds degradation. (DOC) [file pone.0031261.s011.doc]

## Table S9 Genes in hydrocarbon and aromatic compounds degradation

| **Locus_Tag** | **Encoded Protein** | **Func. ID** | **Func. name** | **EC number** |
| --- | --- | --- | --- | --- |
| **Benzoate degradation** | | | | |
| 0118 | 3-hydroxyacyl-CoA dehydrogenase, C-terminal domain family | KO:K00074 | 3-hydroxybutyryl-CoA dehydrogenase | EC:1.1.1.157 |
| 0136 | Acetyltransferase, GNAT family | KO:K00680 |  |  |
| 0202 | Acetyl-CoA acetyltransferase with thiolase domain (Acetoacetyl-CoA thiolase) | KO:K00626 | acetyl-CoA C-acetyltransferase | EC:2.3.1.9 |
| 0381 | Enoyl-CoA hydratase/isomerase family protein | KO:K01692 | enoyl-CoA hydratase | EC:4.2.1.17 |
| 0536 | 3-hydroxyacyl-CoA dehydrogenase (Hdb-1) | KO:K00074 | 3-hydroxybutyryl-CoA dehydrogenase | EC:1.1.1.157 |
| 1104 | Alcohol dehydrogenase, iron-containing superfamily | KO:K00217 | maleylacetate reductase | EC:1.3.1.32 |
| 1105 | Dioxygenase subfamily, putative | KO:K03381 | catechol 1,2-dioxygenase | EC:1.13.11.1 |
| 1481 | Fumarylacetoacetate hydrolase family protein, putative | KO:K01826 | 5-carboxymethyl-2-hydroxymuconate isomerase | EC:5.3.3.10 |
| 1628 | Enoyl-CoA hydratase/isomerase family protein | KO:K01692 | enoyl-CoA hydratase | EC:4.2.1.17 |
| 1858 | Enoyl-CoA hydratase/isomerase family protein | KO:K01692 | enoyl-CoA hydratase | EC:4.2.1.17 |
| 1861 | AMP-dependent synthetase and ligase | KO:K04110 | benzoate-CoA ligase | EC:6.2.1.25 |
| 1877 | Phosphonate metabolim protein, transferase hexapeptide repeat family | KO:K00680 |  |  |
| 1910 | Enoyl-CoA hydratase/isomerase family protein | KO:K01692 | enoyl-CoA hydratase | EC:4.2.1.17 |
| 1943 | Enoyl-CoA hydratase/isomerase family protein | KO:K01692 | enoyl-CoA hydratase | EC:4.2.1.17 |
| 1977 | Extradiol ring-cleavage dioxygenase:Glyoxalase/Bleomycin resistance protein/dioxygenase domain:Glyoxalase I | KO:K00446 | catechol 2,3-dioxygenase | EC:1.13.11.2 |
| 1980 | 5-carboxymethyl-2-hydroxymuconate isomerase | KO:K01826 | 5-carboxymethyl-2-hydroxymuconate isomerase | EC:5.3.3.10 |
| 2155 | Enoyl-CoA hydratase / short chain enoyl-CoA hydratase | KO:K01692 | enoyl-CoA hydratase | EC:4.2.1.17 |
| 2453 | Enoyl-CoA hydratase/isomerase | KO:K01692 | enoyl-CoA hydratase | EC:4.2.1.17 |
| 2470 | Intradiol ring-cleavage dioxygenase:Catechol dioxygenase,N-terminal | KO:K03381 | catechol 1,2-dioxygenase | EC:1.13.11.1 |
| 2513 | Enoyl-CoA hydratase / short chain enoyl-CoA hydratase | KO:K01692 | enoyl-CoA hydratase | EC:4.2.1.17 |
| 2515 | Putative 3-ketoacyl-CoA thiolase (Fatty oxidation complex beta subunit) (Beta-ketothiolase) (Acetyl-CoA acyltransferase) (FadA-like) | KO:K00626 | acetyl-CoA C-acetyltransferase | EC:2.3.1.9 |
| 2714 | 3-oxoadipate enol-lactonase-like and alpha/beta hydrolase suprfamily domains | KO:K01055 | 3-oxoadipate enol-lactonase | EC:3.1.1.24 |
| 2715 | Gamma-carboxymuconolactone decarboxylase protein | KO:K01607 | 4-carboxymuconolactone decarboxylase | EC:4.1.1.44 |
| 2716 | Protocatechuate 3,4-dioxygenase beta chain | KO:K00449 | protocatechuate 3,4-dioxygenase, beta subunit | EC:1.13.11.3 |
| 2717 | Protocatechuate 3,4-dioxygenase, alpha subunit | KO:K00448 | protocatechuate 3,4-dioxygenase, alpha subunit | EC:1.13.11.3 |
| 2720 | Beta-ketothiolase protein | KO:K00626 | acetyl-CoA C-acetyltransferase | EC:2.3.1.9 |
| 2721 | Putative 3-carboxy-cis,cis-muconate cycloisomerase (3-carboxymuconate lactonizing enzyme) | KO:K01857 | 3-carboxy-cis,cis-muconate cycloisomere | EC:5.5.1.2 |
| 2722 | p-hydroxybenzoate hydroxylase transcriptional activator | KO:K00481 | p-hydroxybenzoate 3-monooxygenase | EC:1.14.13.2 |
| 2852 | Metal-dependent hydrolase of the TIM-barrel fold family | KO:K10221 | 2-pyrone-4,6-dicarboxylate lactonase | EC:3.1.1.57 |
| 2858 | Metapyrocatechase; catechol 2,3-dioxygenase | KO:K00446 | catechol 2,3-dioxygenase | EC:1.13.11.2 |
| 2869 | Enoyl-CoA hydratase | KO:K01692 | enoyl-CoA hydratase | EC:4.2.1.17 |
| 3136 | Protocatechuate 4,5-dioxygenase beta chain subunit b protein | KO:K04101 | protocatechuate 4,5-dioxygenase, beta chain | EC:1.13.11.8 |
| 3140 | P-hydroxybenzoate hydroxylase (4-hydroxybenzoate 3-monooxygenase) (PHBH) | KO:K00481 | p-hydroxybenzoate 3-monooxygenase | EC:1.14.13.2 |
| 3176 | Gamma-carboxymuconolactone decarboxylase protein | KO:K01607 | 4-carboxymuconolactone decarboxylase | EC:4.1.1.44 |
| 3188 | Probable catechol 1,2-dioxygenase protein | KO:K03381 | catechol 1,2-dioxygenase | EC:1.13.11.1 |
| 3244 | Putative 3-ketoacyl-CoA thiolase (Fatty oxidation complex beta subunit) (Beta-ketothiolase) (Acetyl-CoA acyltransferase) (FadA-like) | KO:K00626 | acetyl-CoA C-acetyltransferase | EC:2.3.1.9 |
| 3292 | Enoyl-CoA hydratase/isomerase family protein | KO:K01692 | enoyl-CoA hydratase | EC:4.2.1.17 |
| 3431 | Probable acetyltransferase protein | KO:K00680 |  |  |
| 3535 | Thiolase, N-terminal domain subfamily, putative | KO:K00626 | acetyl-CoA C-acetyltransferase | EC:2.3.1.9 |
| 3800 | Enoyl-CoA hydratase, mitochondrial precursor (EC 4.2.1.17) (Shor t chain enoyl-CoA hydratase) (SCEH) (Enoyl-CoA hydratase 1). | KO:K01692 | enoyl-CoA hydratase | EC:4.2.1.17 |
| 3803 | Short-chain dehydrogenase/reductase SDR | KO:K07535 | 2-hydroxycyclohexanecarboxyl-CoA dehydrogenase | EC:1.1.1.- |
| 3810 | Acyl-CoA dehydrogenase domain protein | KO:K04117 | cyclohexanecarboxyl-CoA dehydrogenase | EC:1.3.99.- |
| 3811 | AMP-dependent synthetase and ligase | KO:K04116 | cyclohexanecarboxylate-CoA ligase | EC:6.2.1.- |
| 4239 | GCN5-related N-acetyltransferase | KO:K00680 |  |  |
| 4271 | Acyl-CoA dehydrogenase, C-terminal domain protein | KO:K00252 | glutaryl-CoA dehydrogenase | EC:1.3.99.7 |
| 4332 | Enoyl-CoA hydratase/isomerase family protein | KO:K01692 | enoyl-CoA hydratase | EC:4.2.1.17 |
| p0057 | Acetaldehyde dehydrogenase NahO | KO:K04073 | acetaldehyde dehydrogenase | EC:1.2.1.10 |
| p0059 | 4-hydroxy-2-oxovalerate aldolase NahM | KO:K01666 | 4-hydroxy 2-oxovalerate aldolase | EC:4.1.3.39 |
| **Aminobenzoate degradation** | | | | |
| 0136 | Acetyltransferase, GNAT family | KO:K00680 |  |  |
| 0381 | Enoyl-CoA hydratase/isomerase family protein | KO:K01692 | enoyl-CoA hydratase | EC:4.2.1.17 |
| 0818 | Nitrile hydratase, beta subunit | KO:K01721 | nitrile hydratase | EC:4.2.1.84 |
| 0819 | Nitrile hydratase, alpha subunit | KO:K01721 | nitrile hydratase | EC:4.2.1.84 |
| 1417 | Coenzyme F420-dependent N5 N10-methylene tetrahydromethanopterin reductase and related flavin-dependent oxidoreductase-like protein | KO:K00492 |  |  |
| 1628 | Enoyl-CoA hydratase/isomerase family protein | KO:K01692 | enoyl-CoA hydratase | EC:4.2.1.17 |
| 1790 | Acylphosphatase, putative | KO:K01512 | acylphosphatase | EC:3.6.1.7 |
| 1806 | Oxygenase (Tetracycline 6-hydroxylase) protein | KO:K03380 | phenol 2-monooxygenase | EC:1.14.13.7 |
| 1858 | Enoyl-CoA hydratase/isomerase family protein | KO:K01692 | enoyl-CoA hydratase | EC:4.2.1.17 |
| 1861 | AMP-dependent synthetase and ligase | KO:K04110 | benzoate-CoA ligase | EC:6.2.1.25 |
| 1877 | Phosphonate metabolim protein, transferase hexapeptide repeat family | KO:K00680 |  |  |
| 1910 | Enoyl-CoA hydratase/isomerase family protein | KO:K01692 | enoyl-CoA hydratase | EC:4.2.1.17 |
| 1943 | Enoyl-CoA hydratase/isomerase family protein | KO:K01692 | enoyl-CoA hydratase | EC:4.2.1.17 |
| 2155 | Enoyl-CoA hydratase / short chain enoyl-CoA hydratase | KO:K01692 | enoyl-CoA hydratase | EC:4.2.1.17 |
| 2453 | Enoyl-CoA hydratase/isomerase | KO:K01692 | enoyl-CoA hydratase | EC:4.2.1.17 |
| 2513 | Enoyl-CoA hydratase / short chain enoyl-CoA hydratase | KO:K01692 | enoyl-CoA hydratase | EC:4.2.1.17 |
| 2716 | Protocatechuate 3,4-dioxygenase beta chain | KO:K00449 | protocatechuate 3,4-dioxygenase, beta subunit | EC:1.13.11.3 |
| 2717 | Protocatechuate 3,4-dioxygenase, alpha subunit | KO:K00448 | protocatechuate 3,4-dioxygenase, alpha subunit | EC:1.13.11.3 |
| 2852 | Metal-dependent hydrolase of the TIM-barrel fold family | KO:K10221 | 2-pyrone-4,6-dicarboxylate lactonase | EC:3.1.1.57 |
| 2861 | Oxygenase subunit | KO:K03863 | vanillate monooxygenase | EC:1.14.13.82 |
| 2863 | Vanillate O-demethylase oxygenase, iron-sulfur subunit (Modular protein=subunit from monooxygenase) (VanA-like) | KO:K03862 | vanillate monooxygenase | EC:1.14.13.82 |
| 2869 | Enoyl-CoA hydratase | KO:K01692 | enoyl-CoA hydratase | EC:4.2.1.17 |
| 3136 | Protocatechuate 4,5-dioxygenase beta chain subunit b protein | KO:K04101 | protocatechuate 4,5-dioxygenase, beta chain | EC:1.13.11.8 |
| 3164 | Cytochrome P450-pinF2, plant-inducible | KO:K00517 |  |  |
| 3292 | Enoyl-CoA hydratase/isomerase family protein | KO:K01692 | enoyl-CoA hydratase | EC:4.2.1.17 |
| 3431 | Probable acetyltransferase protein | KO:K00680 |  |  |
| 3800 | Enoyl-CoA hydratase, mitochondrial precursor (EC 4.2.1.17) (Shor t chain enoyl-CoA hydratase) (SCEH) (Enoyl-CoA hydratase 1). | KO:K01692 | enoyl-CoA hydratase | EC:4.2.1.17 |
| 4239 | GCN5-related N-acetyltransferase | KO:K00680 |  |  |
| 4332 | Enoyl-CoA hydratase/isomerase family protein | KO:K01692 | enoyl-CoA hydratase | EC:4.2.1.17 |
| **Fluorobenzoate degradation** | | | | |
| 0818 | Nitrile hydratase, beta subunit | KO:K01721 | nitrile hydratase | EC:4.2.1.84 |
| 0819 | Nitrile hydratase, alpha subunit | KO:K01721 | nitrile hydratase | EC:4.2.1.84 |
| 1104 | Alcohol dehydrogenase, iron-containing superfamily | KO:K00217 | maleylacetate reductase | EC:1.3.1.32 |
| 1105 | Dioxygenase subfamily, putative | KO:K03381 | catechol 1,2-dioxygenase | EC:1.13.11.1 |
| 2470 | Intradiol ring-cleavage dioxygenase:Catechol dioxygenase,N-terminal | KO:K03381 | catechol 1,2-dioxygenase | EC:1.13.11.1 |
| 3188 | Probable catechol 1,2-dioxygenase protein | KO:K03381 | catechol 1,2-dioxygenase | EC:1.13.11.1 |
| 3313 | Dienelactone hydrolase family | KO:K01061 | carboxymethylenebutenolidase | EC:3.1.1.45 |
| [**Chlorocyclohexane and chlorobenzene degradation**](https://img.jgi.doe.gov/cgi-bin/er/main.cgi?section=KeggPathwayDetail&page=koterm&kegg_id=00361&pathway_id=41) | | | | |
| 1104 | Alcohol dehydrogenase, iron-containing superfamily | KO:K00217 | maleylacetate reductase | EC:1.3.1.32 |
| 1105 | Dioxygenase subfamily, putative | KO:K03381 | catechol 1,2-dioxygenase | EC:1.13.11.1 |
| 1417 | Coenzyme F420-dependent N5 N10-methylene tetrahydromethanopterin reductase and related flavin-dependent oxidoreductase-like protein | KO:K00492 |  |  |
| 1806 | Oxygenase (Tetracycline 6-hydroxylase) protein | KO:K03380 | phenol 2-monooxygenase | EC:1.14.13.7 |
| 1977 | Extradiol ring-cleavage dioxygenase:Glyoxalase/Bleomycin resistance protein/dioxygenase domain:Glyoxalase I | KO:K00446 | catechol 2,3-dioxygenase | EC:1.13.11.2 |
| 2470 | Intradiol ring-cleavage dioxygenase:Catechol dioxygenase,N-terminal | KO:K03381 | catechol 1,2-dioxygenase | EC:1.13.11.1 |
| 2858 | Metapyrocatechase; catechol 2,3-dioxygenase | KO:K00446 | catechol 2,3-dioxygenase | EC:1.13.11.2 |
| 2980 | 2-haloacid halidohydrolase Iva | KO:K01560 | 2-haloacid dehalogenase | EC:3.8.1.2 |
| 3188 | Probable catechol 1,2-dioxygenase protein | KO:K03381 | catechol 1,2-dioxygenase | EC:1.13.11.1 |
| 3313 | Dienelactone hydrolase family | KO:K01061 | carboxymethylenebutenolidase | EC:3.1.1.45 |
| **Toluene degradation** | | | | |
| 0184 | Succinate dehydrogenase iron-sulfur subunit | KO:K00240 | succinate dehydrogenase iron-sulfur protein | EC:1.3.99.1 |
| 0185 | Succinate dehydrogenase flavoprotein subunit | KO:K00239 | succinate dehydrogenase flavoprotein subunit | EC:1.3.99.1 |
| 0186 | Succinate dehydrogenase, hydrophobic membrane anchor protein, putative | KO:K00242 | succinate dehydrogenase hydrophobic membrane anchor protein |  |
| 0187 | Succinate dehydrogenase, cytochrome b556 subunit | KO:K00241 | succinate dehydrogenase cytochrome b-556 subunit |  |
| 1104 | Alcohol dehydrogenase, iron-containing superfamily | KO:K00217 | maleylacetate reductase | EC:1.3.1.32 |
| 1105 | Dioxygenase subfamily, putative | KO:K03381 | catechol 1,2-dioxygenase | EC:1.13.11.1 |
| 1417 | Coenzyme F420-dependent N5 N10-methylene tetrahydromethanopterin reductase and related flavin-dependent oxidoreductase-like protein | KO:K00492 |  |  |
| 1806 | Oxygenase (Tetracycline 6-hydroxylase) protein | KO:K03380 | phenol 2-monooxygenase | EC:1.14.13.7 |
| 2470 | Intradiol ring-cleavage dioxygenase:Catechol dioxygenase,N-terminal | KO:K03381 | catechol 1,2-dioxygenase | EC:1.13.11.1 |
| 3188 | Probable catechol 1,2-dioxygenase protein | KO:K03381 | catechol 1,2-dioxygenase | EC:1.13.11.1 |
| 3313 | Dienelactone hydrolase family | KO:K01061 | carboxymethylenebutenolidase | EC:3.1.1.45 |
| 3827 | Fumarate reductase/succinate dehydrogenase flavoprotein-like protein | KO:K00244 | fumarate reductase flavoprotein subunit | EC:1.3.99.1 |
| **Bisphenol degradation** | | | | |
| 0043 | Catalytic LigB subunit of aromatic ring-opening dioxygenase superfamily | KO:K05915 |  |  |
| 0444 | Oxidoreductase, aldo/keto reductase family | KO:K00100 |  |  |
| 1417 | Coenzyme F420-dependent N5 N10-methylene tetrahydromethanopterin reductase and related flavin-dependent oxidoreductase-like protein | KO:K00492 |  |  |
| 2556 | GDP-mannose dehydratase family | KO:K00100 |  |  |
| 3120 | 2,4-dihydroxyacetophenone dioxygenase (Fragment) | KO:K05913 | 2,4-dihydroxyacetophenone dioxygenase [EC:1.13.11.41] | EC:1.13.11.4 |
| 3164 | Cytochrome P450-pinF2, plant-inducible | KO:K00517 |  |  |
| **Dioxin degradation** | | | | |
| 1624 | Monooxygenase, FAD-binding | KO:K00480 | salicylate hydroxylase | EC:1.14.13.1 |
| 4095 | IPB-dioxygenase, ISP large subunit (IpbA1) | KO:K08689 | biphenyl 2,3-dioxygenase | EC:1.14.12.18 |
| 4096 | Biphenyl 2,3-dioxygenase beta subunit | KO:K08689 | biphenyl 2,3-dioxygenase | EC:1.14.12.18 |
| 4097 | Chlorobenzene dioxygenase, ferredoxin | KO:K08689 | biphenyl 2,3-dioxygenase | EC:1.14.12.18 |
| 4098 | Short-chain dehydrogenase/reductase SDR | KO:K08690 | cis-2,3-dihydrobiphenyl-2,3-diol dehydrogenase | EC:1.3.1.56 |
| p0057 | Acetaldehyde dehydrogenase NahO | KO:K04073 | acetaldehyde dehydrogenase | EC:1.2.1.10 |
| p0059 | 4-hydroxy-2-oxovalerate aldolase NahM | KO:K01666 | 4-hydroxy 2-oxovalerate aldolase | EC:4.1.3.39 |
| **Naphthalene degradation** | | | | |
| 0043 | Catalytic LigB subunit of aromatic ring-opening dioxygenase superfamily | KO:K05915 |  |  |
| 0136 | Acetyltransferase, GNAT family | KO:K00680 |  |  |
| 1013 | Alcohol dehydrogenase GroES-like | KO:K00121 | S-(hydroxymethyl)glutathione dehydrogenase / alcohol dehydrogenase | EC:1.1.1.284 1.1.1.1 |
| 1417 | Coenzyme F420-dependent N5 N10-methylene tetrahydromethanopterin reductase and related flavin-dependent oxidoreductase-like protein | KO:K00492 |  |  |
| 1624 | Monooxygenase, FAD-binding | KO:K00480 | salicylate hydroxylase | EC:1.14.13.1 |
| 1877 | Phosphonate metabolim protein, transferase hexapeptide repeat family | KO:K00680 |  |  |
| 1909 | Acyl-CoA dehydrogenase family member 8, mitochondrial precursor (EC 1.3.99.-) (ACAD-8) (Isobutyryl-CoA dehydrogenase) (Activator- recruited cofactor 42 kDa component) (ARC42). | KO:K00257 |  |  |
| 2138 | Acyl-CoA dehydrogenase domain protein | KO:K00257 |  |  |
| 2719 | Similar to glutaconate CoA-transferase chain B | KO:K01041 |  |  |
| 2872 | Acyl-CoA dehydrogenase | KO:K00257 |  |  |
| 3241 | Acyl-CoA dehydrogenase, C-terminal domain protein | KO:K00257 |  |  |
| 3431 | Probable acetyltransferase protein | KO:K00680 |  |  |
| 3497 | Acyl-CoA dehydrogenase domain protein | KO:K00257 |  |  |
| 3562 | Dehydrogenase, (Iron-containing alcohol dehydrogenase, 4-hydroxybutyrate dehydrogenase, methanol dehydrogenase) | KO:K00001 | alcohol dehydrogenase | EC:1.1.1.1 |
| 4239 | GCN5-related N-acetyltransferase | KO:K00680 |  |  |
| **Metabolism of xenobiotics by cytochrome P450** | | | | |
| 1108 | 4,5-dihydroxyphthalate decarboxylase | KO:K04102 | 4,5-dihydroxyphthalate decarboxylase | EC:4.1.1.55 |
| 1417 | Coenzyme F420-dependent N5 N10-methylene tetrahydromethanopterin reductase and related flavin-dependent oxidoreductase-like protein | KO:K00492 |  |  |
| 1624 | Monooxygenase, FAD-binding | KO:K00480 | salicylate hydroxylase | EC:1.14.13.1 |
| 2716 | Protocatechuate 3,4-dioxygenase beta chain | KO:K00449 | protocatechuate 3,4-dioxygenase, beta subunit | EC:1.13.11.3 |
| 2717 | Protocatechuate 3,4-dioxygenase, alpha subunit | KO:K00448 | protocatechuate 3,4-dioxygenase, alpha subunit | EC:1.13.11.3 |
| 3136 | Protocatechuate 4,5-dioxygenase beta chain subunit b protein | KO:K04101 | protocatechuate 4,5-dioxygenase, beta chain | EC:1.13.11.8 |
| 3164 | Cytochrome P450-pinF2, plant-inducible | KO:K00517 |  |  |
| 4082 | Dihydrodipicolinate synthetase | KO:K11949 | 4-(2-carboxyphenyl)-2-oxobut-3-enoate aldolase | EC:4.1.2.34 |
| 4105 | Dihydrodipicolinate synthetase | KO:K11949 | 4-(2-carboxyphenyl)-2-oxobut-3-enoate aldolase | EC:4.1.2.34 |
| **Alkane degradation** | | | | |
| 0064 | Oxidoreductase, short chain dehydrogenase/reductase family | KO:K00208 | enoyl-[acyl-carrier protein] reductase I | EC:1.3.1.9; |
| 0112 | Oxidoreductase, short chain dehydrogenase/reductase family |  |  |  |
| 0174 | Oxidoreductase, zinc-binding dehydrogenase family |  |  | EC:1.6.5.5; |
| 0389 | Oxidoreductase, zinc-binding dehydrogenase family |  |  |  |
| 0890 | Short-chain dehydrogenase/reductase SDR |  |  |  |
| 1013 | Alcohol dehydrogenase GroES-like | KO:K00121 | S-(hydroxymethyl)glutathione dehydrogenase / alcohol dehydrogenase | EC:1.1.1.284;EC:1.1.1.1; |
| 1016 | Methanol dehydrogenase large subunit-like protein |  |  |  |
| 1066 | Oxidoreductase, short-chain dehydrogenase/reductase family | KO:K00059 | 3-oxoacyl-[acyl-carrier protein] reductase | EC:1.1.1.100; |
| 1104 | Alcohol dehydrogenase, iron-containing superfamily | KO:K00217 | maleylacetate reductase | EC:1.3.1.32; |
| 1130 | Short-chain dehydrogenase/reductase SDR | KO:K00059 | 3-oxoacyl-[acyl-carrier protein] reductase | EC:1.1.1.100; |
| 1473 | Aldehyde oxidase and xanthine dehydrogenase, molybdopterin binding domain, putative | KO:K03520 | carbon-monoxide dehydrogenase large subunit | EC:1.2.99.2; |
| 1768 | Short-chain dehydrogenase/reductase SDR |  |  |  |
| 1856 | Short-chain dehydrogenase/reductase SDR |  |  |  |
| 2151 | Aldehyde dehydrogenase (NAD+) |  |  |  |
| 2341 | Short-chain dehydrogenase/reductase SDR | KO:K00059 | 3-oxoacyl-[acyl-carrier protein] reductase | EC:1.1.1.100; |
| 2533 | Short-chain dehydrogenase/reductase SDR | KO:K00059 | 3-oxoacyl-[acyl-carrier protein] reductase | EC:1.1.1.100; |
| 2677 | Short-chain dehydrogenase/reductase SDR |  |  |  |
| 2764 | Short-chain dehydrogenase/reductase SDR |  |  |  |
| 2814 | Short-chain dehydrogenase/reductase SDR |  |  |  |
| 3115 | Short-chain dehydrogenase/reductase SDR | KO:K00059 | 3-oxoacyl-[acyl-carrier protein] reductase | EC:1.1.1.100; |
| 3121 | Short-chain dehydrogenase/reductase SDR |  |  |  |
| 3155 | Aldehyde dehydrogenase (NAD) family protein |  |  |  |
| 3166 | Short-chain dehydrogenase/reductase SDR |  |  |  |
| 3189 | Short-chain dehydrogenase/reductase SDR |  |  |  |
| 3456 | Aldehyde dehydrogenase (NAD) family protein |  |  | EC:1.2.1.68; |
| 3527 | Aldehyde dehydrogenase 2B4 |  |  |  |
| 3561 | PREDICTED: aldehyde dehydrogenase 1A1-like |  |  |  |
| 3562 | Dehydrogenase, (Iron-containing alcohol dehydrogenase, 4-hydroxybutyrate dehydrogenase, methanol dehydrogenase) |  |  |  |
| 3803 | Short-chain dehydrogenase/reductase SDR | KO:K07535 | 2-hydroxycyclohexanecarboxyl-CoA dehydrogenase | EC:1.1.1.- |
| 3812 | Short-chain dehydrogenase/reductase SDR |  |  |  |
| 3946 | Aldehyde dehydrogenase |  |  |  |
| 4158 | Short-chain dehydrogenase/reductase SDR |  |  |  |
| 1417 | Coenzyme F420-dependent N5 N10-methylene tetrahydromethanopterin reductase and related flavin-dependent oxidoreductase-like protein |  |  |  |
| 2655 | Oxidoreductase protein |  |  |  |
| 2670 | Luciferase-like monooxygenase superfamily |  |  |  |
| 1954 | Cytochrome P450 |  |  |  |
| 3164 | Cytochrome P450-pinF2, plant-inducible |  |  |  |
| 4100 | Putative cytochrome p450-like enzyme |  |  |  |
