# Supplementary material for: The Genome Sequence of Polymorphum gilvum SL003B-26A1T Reveals Its Genetic Basis for Crude Oil Degradation and Adaptation to the Saline Soil
Source: PLoS One. 2012 Feb 16;7(2):e31261. doi: 10.1371/journal.pone.0031261 (PMC3281065; doi:10.1371/journal.pone.0031261)
Supplement: Table S10 — Genes in signal transduction (COG category T). (DOC) [file pone.0031261.s012.doc]

## Table S10 Genes in signal transduction (COG category T)

| **Locus_Tag** | **Name** | **COG** |
| --- | --- | --- |
| 0005 | Diguanylate cyclase/phosphodiesterase with PAS/PAC sensor(S) | COG5001 |
| 0016 | Methyl-accepting chemotaxis sensory transducer | COG0840 |
| 0018 | Response regulator receiver domain protein (CheY-like) | COG4567 |
| 0019 | ATPase, histidine kinase-, DNA gyrase B-, and HSP90-like domain protein | COG0642 |
| 0032 | ATPase, histidine kinase-, DNA gyrase B-, and HSP90-like domain protein | COG0642 |
| 0127 | Response regulator receiver (CheY-like protein) | COG2204 |
| 0155 | Putative cAMP-dependent kinase protein | COG0664 |
| 0156 | Putative transcription regulator protein | COG0745 |
| 0179 | Methyl-accepting chemotaxis protein | COG0840 |
| 0211 | Diguanylate cyclase/phosphodiesterase with PAS/PAC sensor(S) | COG5001 |
| 0220 | Membrane GTPase involved in stress response | COG1217 |
| 0225 | TSPO-like protein (Fragment) | COG3476 |
| 0235 | ATPase, histidine kinase-, DNA gyrase B-, and HSP90-like domain protein | COG3437,COG0642 |
| 0239 | Probable tyrosine phosphatase protein | COG0394 |
| 0326 | Chemotaxis sensory transducer | COG0840 |
| 0344 | BolA-like protein | COG0271 |
| 0354 | Signal transduction histidine kinase | COG3706 |
| 0367 | Cyclic diguanylate phosphodiesterase domain protein | COG2200 |
| 0410 | two component transcriptional regulator, LuxR family protein | COG3706 |
| 0411 | ATPase, histidine kinase-, DNA gyrase B-, and HSP90-like domain protein | COG3437,COG0642 |
| 0451 | Chemotaxis sensory transducer (Fragment) | COG0840 |
| 0456 | Adenylate cyclase protein | COG2114 |
| 0474 | Phosphoenolpyruvate-protein phosphotransferase, PtsP | COG3605 |
| 0510 | PEMK-like protein | COG2337 |
| 0538 | Two-component system sensor protein | COG0642 |
| 0539 | Two component transcriptional regulator, winged helix family | COG0745 |
| 0586 | Transcriptional regulator, Crp/Fnr family | COG0664 |
| 0607 | UspA domain protein | COG0589 |
| 0643 | Conjugation factor synthetase; TraI | COG3916 |
| 0692 | PEMK-like protein | COG2337 |
| 0702 | Conjugation factor synthetase; TraI | COG3916 |
| 0760 | Probable two-component sensor histidine kinase protein | COG4191 |
| 0761 | Two component, sigma54 specific, transcriptional regulator, Fis family | COG2204 |
| 0774 | Putative adenylate cyclase transmembrane protein | COG2114 |
| 0799 | Histidine kinase, HAMP region:Bacterial chemotaxis sensory transducer | COG0840 |
| 0821 | Response regulator containing a CheY-like receiver domain and an HTH DNA-binding domain | COG2197 |
| 0822 | Signal transduction histidine kinase | COG4564 |
| 0829 | DNA-binding response regulator | COG0745 |
| 0830 | PAS | COG0642 |
| 0859 | Response regulator receiver domain protein (CheY-like) | COG2204 |
| 0870 | Sigma-54 factor interaction domain-containing protein | COG2204 |
| 0919 | Methyl-accepting chemotaxis sensory transducer | COG0840 |
| 0942 | Putative arsenate reductase; arsC-like protein | COG0394 |
| 0948 | CheA-like signal transduction histidine kinase | COG0643 |
| 0949 | Probable purine-binding chemotaxis protein | COG0835 |
| 0950 | Response regulator receiver domain protein (CheY-like) | COG3706 |
| 0951 | Chemotaxis response regulator protein-glutamate methylesterase | COG2201 |
| 0952 | CheR methyltransferase, SAM binding domain protein | COG1352 |
| 0954 | Two-component transcriptional regulator | COG0745 |
| 1003 | Two component transcriptional regulator, LuxR family | COG2197 |
| 1005 | Histidine kinase, putative | COG0642 |
| 1006 | Response regulator receiver:ATP-binding region, ATPase-like:Histidine kinase, HAMP region:Histidine | COG0642 |
| 1007 | COG0745: Response regulators consisting of a CheY-like receiver domain and a winged-helix DNA-binding domain | COG0745 |
| 1018 | Extracellular solute-binding protein, family 3 | COG0834 |
| 1099 | UspA domain protein | COG0589 |
| 1147 | COG2204: Response regulator containing CheY-like receiver, AAA-t ype ATPase, and DNA-binding domains | COG2204 |
| 1169 | Methyl-accepting chemotaxis sensory transducer | COG0840 |
| 1190 | Conserved hypothetical exported protein | COG3290 |
| 1202 | ATPase, histidine kinase-, DNA gyrase B-, and HSP90-like domain protein | COG0642 |
| 1203 | Putative transcription regulator protein | COG0745 |
| 1237 | Cyclic diguanylate phosphodiesterase domain protein | COG2199 |
| 1314 | Methyl-accepting chemotaxis protein | COG0840 |
| 1316 | Diguanylate cyclase with PAS/PAC sensor | COG3706 |
| 1331 | Cyclic diguanylate phosphodiesterase domain protein | COG5001 |
| 1378 | Putative uncharacterized protein | COG0589 |
| 1384 | Putative diguanylate cyclase (GGDEF)/phosphodiesterase (EAL), with PAS sensor, signal transduction histidine kinase and methyl-accepting chemotaxis domains; putative membrane-associated protein | COG5001 |
| 1421 | Histidine kinase, HAMP region:Bacterial chemotaxis sensory transducer | COG0840 |
| 1462 | Methyl-accepting chemotaxis sensory transducer | COG0840 |
| 1488 | Methyl-accepting chemotaxis sensory transducer | COG0840 |
| 1489 | Putative uncharacterized protein | COG3143 |
| 1499 | Hpt domain protein | COG2198 |
| 1506 | Universal stress protein family, putative | COG0589 |
| 1514 | Phosphohistidine Phosphatase, SixA | COG2062 |
| 1534 | transcriptional regulator, TraR/DksA family protein | COG1734 |
| 1552 | Sensor kinase | COG0642 |
| 1562 | Ion transport protein | COG0664 |
| 1575 | Regulator of penicillin binding proteins and beta lactamase transcription | COG0271 |
| 1615 | Cyclic diguanylate phosphodiesterase domain protein | COG5001 |
| 1638 | Inosine-5prime-monophosphate dehydrogenase protein | COG3448 |
| 1652 | Hpt domain protein | COG0643 |
| 1675 | DnaK suppressor protein | COG1734 |
| 1765 | Methyl-accepting chemotaxis protein signaling domain | COG0840 |
| 1789 | Sensory box/response regulator | COG2199 |
| 1801 | COG3706: Response regulator containing a CheY-like receiver doma in and a GGDEF domain | COG3706 |
| 1810 | Diguanylate cyclase with GGDEF motif; putative membrane protein | COG2199 |
| 1816 | Adenylate and Guanylate cyclase catalytic domain protein | COG2114 |
| 1841 | COG0784: FOG: CheY-like receiver | COG0784 |
| 1899 | GTP pyrophosphokinase (ATP:GTP 3-pyrophosphotransferase) (PpGpp synthetase I) ((P)ppGpp synthetase) | COG0317 |
| 1973 | CHASE2 domain family | COG2114 |
| 1993 | Putative uncharacterized protein | COG3629 |
| 2004 | Amino acid ABC transporter, periplasmic amino acid-binding protein | COG0834 |
| 2015 | Transcriptional activator (Anti-sigma factor) protein | COG3806 |
| 2023 | Universal stress protein uspA | COG0589 |
| 2110 | COG1974: SOS-response transcriptional repressors (RecA-mediated autopeptidases) | COG1974 |
| 2122 | Bll1335 protein | COG4585 |
| 2123 | Two component transcriptional regulator, LuxR family | COG2197 |
| 2234 | Putative uncharacterized protein | COG3706 |
| 2302 | Sigma-54 factor interaction domain-containing protein | COG2204 |
| 2303 | ATPase, histidine kinase-, DNA gyrase B-, and HSP90-like domain protein | COG5000 |
| 2307 | COG2204: Response regulator containing CheY-like receiver, AAA-t ype ATPase, and DNA-binding domains | COG2204 |
| 2308 | ATPase, histidine kinase-, DNA gyrase B-, and HSP90-like domain protein | COG3852 |
| 2335 | Two component, sigma54 specific, transcriptional regulator, Fis family | COG2204 |
| 2356 | Putative diguanylate cyclase (GGDEF) | COG3706 |
| 2367 | Response regulator receiver domain protein | COG3437 |
| 2371 | Cyclic diguanylate phosphodiesterase domain protein | COG4943 |
| 2420 | Transcriptional regulator, TraR/DksA family protein | COG1734 |
| 2427 | Tyrosine-protein phosphatase, putative | COG2365 |
| 2510 | DNA polymerase III, epsilon subunit | COG2905 |
| 2534 | Extracellular solute-binding protein, family 3 | COG0834 |
| 2560 | UspA domain protein | COG0589 |
| 2580 | Two component transcriptional regulator, winged helix family | COG0745 |
| 2581 | Periplasmic sensor signal transduction histidine kinase | COG0642 |
| 2584 | Putative phosphoenolpyruvate-protein phosphotransferase | COG3605 |
| 2623 | Putative universal stress protein, UspA-like protein | COG0589 |
| 2678 | Adenylate and Guanylate cyclase catalytic domain protein | COG2114 |
| 2680 | Putative transcriptional regulator protein | COG0664 |
| 2749 | Response regulator receiver domain protein (CheY-like) | COG2204 |
| 2754 | GGDEF domain protein | COG2199 |
| 2758 | Methyl-accepting chemotaxis sensory transducer | COG0840 |
| 2761 | Methyl-accepting chemotaxis sensory transducer | COG0840 |
| 2800 | CHASE2 domain family | COG5001 |
| 2801 | Putative uncharacterized protein | COG3712 |
| 2818 | ABC transporter, substrate binding protein (Amino acid) | COG0834 |
| 2826 | ATPase, histidine kinase-, DNA gyrase B-, and HSP90-like domain protein | COG0642 |
| 2891 | Adenylate cyclase 1 | COG2114 |
| 2898 | Multi-sensor signal transduction histidine kinase | COG0642 |
| 2899 | Rhodopsin-like GPCR superfamily:Glutamate-ammonia ligase adenylyltransferase | COG1391 |
| 2900 | ATPase, histidine kinase-, DNA gyrase B-, and HSP90-like domain protein | COG0642 |
| 2901 | COG0745: Response regulators consisting of a CheY-like receiver domain and a winged-helix DNA-binding domain | COG0745 |
| 2912 | ATPase, histidine kinase-, DNA gyrase B-, and HSP90-like domain protein | COG0642 |
| 2913 | Response regulators consisting of a CheY-like receiver domain and a winged-helix DNA-binding domain | COG0745 |
| 2948 | Sensor histidine kinase with a PAS domain | COG0642 |
| 2963 | Diguanylate cyclase with PAS/PAC sensor | COG3706 |
| 2971 | Transcriptional regulator, Nnr-like | COG0664 |
| 2983 | Transcriptional regulatory protein | COG2197 |
| 3000 | EF hand domain protein | COG5126 |
| 3001 | CheY-like response regulator | COG0745 |
| 3002 | ATPase, histidine kinase-, DNA gyrase B-, and HSP90-like domain protein | COG0642 |
| 3017 | Carbon monoxide dehydrogenase operon C protein | COG3300 |
| 3052 | Methyl-accepting chemotaxis sensory transducer | COG0840 |
| 3053 | Diguanylate cyclase/phosphodiesterase with PAS/PAC and GAF sensor(S) | COG5001 |
| 3102 | Putative arsenate reductase (ArsC-like) | COG0394 |
| 3141 | SENSORY TRANSDUCTION HISTIDINE KINASE | COG2199 |
| 3150 | ABC transporter substrate-binding protein | COG0834 |
| 3205 | Response regulator receiver domain protein (CheY-like) | COG3437 |
| 3216 | Transcriptional regulator, Crp/Fnr family protein | COG0664 |
| 3217 | Tetrathionate reductase complex: response regulator | COG4566 |
| 3218 | Two component transcriptional regulator, LuxR family protein | COG4566 |
| 3219 | Multi-sensor signal transduction histidine kinase | COG4191 |
| 3223 | Two component transcriptional regulator, LuxR family protein | COG2197 |
| 3267 | ABC transporter, periplasmic amino acid-binding protein | COG0834 |
| 3275 | Phosphate regulon transcriptional regulatory protein | COG0745 |
| 3281 | ATPase, histidine kinase-, DNA gyrase B-, and HSP90-like domain protein | COG5002 |
| 3385 | Diguanylate cyclase/phosphodiesterase with PAS/PAC-like protein | COG5001 |
| 3415 | Putative diguanylate cyclase (GGDEF domain) | COG2199 |
| 3417 | Putative transmembrane GGDEF sensory box protein | COG2199 |
| 3423 | Putative diguanylate cyclase/phosphodiesterase (GGDEF &amp; EAL domains) with PAS/PAC sensor | COG5001 |
| 3438 | Chemotaxis protein | COG3143 |
| 3439 | Response regulator receiver (CheY-like protein) | COG2204 |
| 3450 | Cyclic diguanylate phosphodiesterase domain protein | COG2200 |
| 3451 | Sigma-54 interaction domain family | COG2204 |
| 3460 | Cyclic nucleotide-binding:Bacterial regulatory protein, Crp | COG0664 |
| 3470 | Putative uncharacterized protein | COG3143 |
| 3499 | Universal stress protein family | COG0589 |
| 3508 | N-acetylmuramoyl-L-alanine amidase, family 3 | COG3103 |
| 3512 | PAS domain protein | COG0642 |
| 3537 | Response regulator receiver domain protein | COG3437 |
| 3565 | Adenylate/guanylate cyclase | COG2114 |
| 3582 | Cyclic diguanylate phosphodiesterase domain protein | COG5001 |
| 3629 | ATPase, histidine kinase-, DNA gyrase B-, and HSP90-like domain protein | COG0642 |
| 3642 | Type IV pilus assembly protein PilZ | COG0840 |
| 3709 | Methyl-accepting chemotaxis sensory transducer | COG0840 |
| 3710 | Histidine kinase, HAMP region:Bacterial chemotaxis sensory transducer | COG0840 |
| 3720 | Methyl-accepting chemotaxis sensory transducer | COG0840 |
| 3723 | Methyl-accepting chemotaxis sensory transducer | COG0840 |
| 3724 | Chemotaxis sensory transducer | COG2202 |
| 3742 | Methyl-accepting chemotaxis receptor/sensory transducer | COG0840 |
| 3819 | Response regulator of hydrogenase 3 activity (Sensor HydH) | COG3829 |
| 3847 | COG0745: Response regulators consisting of a CheY-like receiver domain and a winged-helix DNA-binding domain | COG0745 |
| 3931 | Phosphoenolpyruvate-dependent sugar phosphotransferase system, EIIA 2:PTS IIA-like nitrogen-regulatory protein PtsN | COG1762 |
| 3941 | Putative transcriptional regulator, LuxR family protein | COG2197 |
| 3954 | Putative transcriptional regulator, Crp/Fnr family | COG0664 |
| 4028 | Two component transcriptional regulator, winged helix family | COG0745 |
| 4029 | Periplasmic sensor signal transduction histidine kinase | COG0642 |
| 4135 | Cyclic diguanylate phosphodiesterase domain protein | COG2199 |
| 4137 | Cyclic diguanylate phosphodiesterase domain protein | COG2200 |
| 4166 | Sensory transduction histidine kinase | COG3614 |
| 4200 | Alkaline phosphatase synthesis sensor protein phor | COG0642 |
| 4204 | COG1493: Serine kinase of the HPr protein, regulates carbohydrat e metabolism | COG1493 |
| 4205 | ATPase, histidine kinase-, DNA gyrase B-, and HSP90-like domain protein | COG0642 |
| 4206 | Response regulator consisting of a CheY-like receiver domain and a winged-helix DNA-binding domain | COG0745 |
| 4240 | Protein-tyrosine phosphatase, low molecular weight | COG0394 |
| 4291 | GGDEF family protein | COG2199 |
| 4313 | Putative enzyme with nucleoside triphosphate hydrolase domain | COG1702 |
| 4320 | Universal stress protein family, putative | COG0589 |
| p0002 | Response regulator receiver protein | COG2197 |
| p0045 | Acyl-homoserine-lactone synthase | COG3916 |
