# Supplementary material for: The Genome Sequence of Polymorphum gilvum SL003B-26A1T Reveals Its Genetic Basis for Crude Oil Degradation and Adaptation to the Saline Soil
Source: PLoS One. 2012 Feb 16;7(2):e31261. doi: 10.1371/journal.pone.0031261 (PMC3281065; doi:10.1371/journal.pone.0031261)
Supplement: Table S11 — Genes in two component systems (pathways via KO terms). (DOC) [file pone.0031261.s013.doc]

## Table S11 Genes in two component systems (pathways via KO terms)

| **Locus_Tag** | **Product Name** | **Func ID** | **Func Name** |
| --- | --- | --- | --- |
| 0001 | Chromosomal replication initiator protein dnaA | KO:K02313 | chromosomal replication initiator protein |
| 0016 | Methyl-accepting chemotaxis sensory transducer | KO:K03406 | methyl-accepting chemotaxis protein |
| 0138 | HpcH/HpaI aldolase/citrate lyase family | KO:K01644 | citrate lyase subunit beta / citryl-CoA lyase [EC:4.1.3.6 4.1.3.34] |
| 0202 | Acetyl-CoA acetyltransferase with thiolase domain (Acetoacetyl-CoA thiolase) | KO:K00626 | acetyl-CoA C-acetyltransferase [EC:2.3.1.9] |
| 0401 | Transcriptional regulator, LuxR family | KO:K07782 | LuxR family transcriptional regulator |
| 0490 | Flagellin-like protein | KO:K02406 | flagellin |
| 0530 | Citrate lyase, beta subunit, putative | KO:K01644 | citrate lyase subunit beta / citryl-CoA lyase [EC:4.1.3.6 4.1.3.34] |
| 0538 | Two-component system sensor protein | KO:K07649 | two-component system, OmpR family, sensor histidine kinase TctE [EC:2.7.13.3] |
| 0539 | Two component transcriptional regulator, winged helix family |  |  |
| 0701 | Putative N-acyl homoserine lactone transcriptional regulator, LuxR-like | KO:K07782 | LuxR family transcriptional regulator |
| 0760 | Probable two-component sensor histidine kinase protein |  |  |
| 0761 | Two component, sigma54 specific, transcriptional regulator, Fis family | KO:K10126 | two-component system, NtrC family, C4-dicarboxylate transport response regulator DctD |
| 0762 | TRAP transporter solute receptor, DctP family protein | KO:K11688 | C4-dicarboxylate-binding protein DctP |
| 0763 | TRAP-type C4-dicarboxylate transport system, small permease component | KO:K11689 | C4-dicarboxylate transporter, DctQ subunit |
| 0764 | TRAP-type C4-dicarboxylate transport system, large permease component | KO:K11690 | C4-dicarboxylate transporter, DctM subunit |
| 0842 | MotA/TolQ/ExbB proton channel | KO:K02556 | chemotaxis protein MotA |
| 0859 | Response regulator receiver domain protein (CheY-like) | KO:K13589 | two-component system, cell cycle response regulator CpdR |
| 0947 | Putative uncharacterized protein | KO:K13588 | histidine phosphotransferase ChpT |
| 0948 | CheA-like signal transduction histidine kinase | KO:K03407 | two-component system, chemotaxis family, sensor kinase CheA [EC:2.7.13.3] |
| 0949 | Probable purine-binding chemotaxis protein | KO:K03408 | purine-binding chemotaxis protein CheW |
| 0950 | Response regulator receiver domain protein (CheY-like) | KO:K03413 | two-component system, chemotaxis family, response regulator CheY |
| 0951 | Chemotaxis response regulator protein-glutamate methylesterase | KO:K03412 | two-component system, chemotaxis family, response regulator CheB [EC:3.1.1.61] |
| 0952 | CheR methyltransferase, SAM binding domain protein | KO:K00575 | chemotaxis protein methyltransferase CheR [EC:2.1.1.80] |
| 0954 | Two-component transcriptional regulator | KO:K13584 | two-component system, cell cycle response regulator CtrA |
| 1067 | Citrate lyase, beta subunit protein | KO:K01644 | citrate lyase subunit beta / citryl-CoA lyase [EC:4.1.3.6 4.1.3.34] |
| 1117 | Tricarboxylate transporter family protein | KO:K07793 | putative tricarboxylic transport membrane protein |
| 1169 | Methyl-accepting chemotaxis sensory transducer | KO:K03406 | methyl-accepting chemotaxis protein |
| 1202 | ATPase, histidine kinase-, DNA gyrase B-, and HSP90-like domain protein | KO:K07638 | two-component system, OmpR family, osmolarity sensor histidine kinase EnvZ [EC:2.7.13.3] |
| 1203 | Putative transcription regulator protein | KO:K07659 | two-component system, OmpR family, phosphate regulon response regulator OmpR |
| 1462 | Methyl-accepting chemotaxis sensory transducer | KO:K03406 | methyl-accepting chemotaxis protein |
| 1552 | Sensor kinase | KO:K13587 | two-component system, cell cycle sensor histidine kinase and response regulator CckA [EC:2.7.13.3] |
| 1841 | CheY-like receiver | KO:K11443 | two-component system, cell cycle response regulator DivK |
| 2058 | Glutamine synthetase 1 | KO:K01915 | glutamine synthetase [EC:6.3.1.2] |
| 2059 | Regulatory protein, P-II 2, for nitrogen assimilation by glutamine synthetase, regulates GlnL (NRII) and GlnE (ATase) | KO:K04751 | nitrogen regulatory protein P-II 1 |
| 2239 | HpcH/HpaI aldolase/citrate lyase family, putative | KO:K01644 | citrate lyase subunit beta / citryl-CoA lyase [EC:4.1.3.6 4.1.3.34] |
| 2302 | Sigma-54 factor interaction domain-containing protein | KO:K13599 | two-component system, NtrC family, nitrogen regulation response regulator NtrX |
| 2303 | ATPase, histidine kinase-, DNA gyrase B-, and HSP90-like domain protein | KO:K13598 | two-component system, NtrC family, nitrogen regulation sensor histidine kinase NtrY [EC:2.7.13.3] |
| 2307 | COG2204: Response regulator containing CheY-like receiver, AAA-t ype ATPase, and DNA-binding domains | KO:K07712 | two-component system, NtrC family, nitrogen regulation response regulator GlnG |
| 2308 | ATPase, histidine kinase-, DNA gyrase B-, and HSP90-like domain protein | KO:K07708 | two-component system, NtrC family, nitrogen regulation sensor histidine kinase GlnL [EC:2.7.13.3] |
| 2515 | Putative 3-ketoacyl-CoA thiolase (Fatty oxidation complex beta subunit) (Beta-ketothiolase) (Acetyl-CoA acyltransferase) (FadA-like) | KO:K00626 | acetyl-CoA C-acetyltransferase [EC:2.3.1.9] |
| 2720 | Beta-ketothiolase protein | KO:K00626 | acetyl-CoA C-acetyltransferase [EC:2.3.1.9] |
| 2758 | Methyl-accepting chemotaxis sensory transducer | KO:K03406 | methyl-accepting chemotaxis protein |
| 2826 | ATPase, histidine kinase-, DNA gyrase B-, and HSP90-like domain protein | KO:K07716 | two-component system, cell cycle sensor histidine kinase PleC [EC:2.7.13.3] |
| 2898 | Multi-sensor signal transduction histidine kinase | KO:K07716 | two-component system, cell cycle sensor histidine kinase PleC [EC:2.7.13.3] |
| 3244 | Putative 3-ketoacyl-CoA thiolase (Fatty oxidation complex beta subunit) (Beta-ketothiolase) (Acetyl-CoA acyltransferase) (FadA-like) | KO:K00626 | acetyl-CoA C-acetyltransferase [EC:2.3.1.9] |
| 3275 | Phosphate regulon transcriptional regulatory protein | KO:K07657 | two-component system, OmpR family, phosphate regulon response regulator PhoB |
| 3280 | ABC-type phosphate transport system periplasmic component-like protein | KO:K02040 | phosphate transport system substrate-binding protein |
| 3281 | ATPase, histidine kinase-, DNA gyrase B-, and HSP90-like domain protein | KO:K07636 | two-component system, OmpR family, phosphate regulon sensor histidine kinase PhoR [EC:2.7.13.3] |
| 3439 | Response regulator receiver (CheY-like protein) | KO:K03413 | two-component system, chemotaxis family, response regulator CheY |
| 3523 | Glutamine synthetase, putative | KO:K01915 | glutamine synthetase [EC:6.3.1.2] |
| 3525 | Glutamine synthetase, catalytic domain, putative | KO:K01915 | glutamine synthetase [EC:6.3.1.2] |
| 3535 | Thiolase, N-terminal domain subfamily, putative | KO:K00626 | acetyl-CoA C-acetyltransferase [EC:2.3.1.9] |
| 3558 | Glutamine synthetase, catalytic domain, putative | KO:K01915 | glutamine synthetase [EC:6.3.1.2] |
| 3710 | Histidine kinase, HAMP region:Bacterial chemotaxis sensory transducer | KO:K03406 | methyl-accepting chemotaxis protein |
| 3742 | Methyl-accepting chemotaxis receptor/sensory transducer | KO:K03406 | methyl-accepting chemotaxis protein |
| 3827 | Fumarate reductase/succinate dehydrogenase flavoprotein-like protein | KO:K00244 | fumarate reductase flavoprotein subunit [EC:1.3.99.1] |
| 3853 | Chemotaxis transmembrane protein | KO:K02556 | chemotaxis protein MotA |
| 3882 | Flagellin-like protein | KO:K02406 | flagellin |
| 3933 | RNA polymerase sigma-54 factor | KO:K03092 | RNA polymerase sigma-54 factor |
| 3965 | Flagellin-like | KO:K02406 | flagellin |
| 4249 | UTP-GlnB uridylyltransferase, GlnD | KO:K00990 | [protein-PII] uridylyltransferase [EC:2.7.7.59] |
