# Supplementary material for: The Genome Sequence of Polymorphum gilvum SL003B-26A1T Reveals Its Genetic Basis for Crude Oil Degradation and Adaptation to the Saline Soil
Source: PLoS One. 2012 Feb 16;7(2):e31261. doi: 10.1371/journal.pone.0031261 (PMC3281065; doi:10.1371/journal.pone.0031261)
Supplement: Table S12 — Genes in cell motility (COG category N). (DOC) [file pone.0031261.s014.doc]

## Table S12 Genes in cell motility (COG category N)

| **Locus_Tag** | **Name** | **COG** |
| --- | --- | --- |
| 0016 | Methyl-accepting chemotaxis sensory transducer | COG0840 |
| 0179 | Methyl-accepting chemotaxis protein | COG0840 |
| 0238 | Protein required for attachment to host cells-like | COG5622 |
| 0284 | Conjugal transfer protein TrbB | COG5268 |
| 0326 | Chemotaxis sensory transducer | COG0840 |
| 0451 | Chemotaxis sensory transducer (Fragment | COG0840 |
| 0489 | Flagellar hook-basal body protein | COG1749 |
| 0490 | Flagellin-like protein | COG1344 |
| 0799 | Histidine kinase, HAMP region:Bacterial chemotaxis sensory transducer | COG0840 |
| 0841 | OmpA family protein | COG1360 |
| 0842 | MotA/TolQ/ExbB proton channel | COG1291 |
| 0880 | Probable O-linked GlcNAc transferase protein | COG3063 |
| 0898 | OmpA family protein | COG1360 |
| 0919 | Methyl-accepting chemotaxis sensory transducer | COG0840 |
| 0948 | CheA-like signal transduction histidine kinase | COG0643 |
| 0949 | Probable purine-binding chemotaxis protein | COG0835 |
| 0951 | Chemotaxis response regulator protein-glutamate methylesterase | COG2201 |
| 0952 | CheR methyltransferase, SAM binding domain protein | COG1352 |
| 0955 | H+-transporting two-sector ATPase FliI | COG1157 |
| 1141 | Flagellar hook capping protein | COG1843 |
| 1143 | Flagellar M-ring protein | COG1766 |
| 1144 | Flagellar motor switch protein FliG | COG1536 |
| 1145 | Flagellar assembly protein H | COG1317 |
| 1146 | Flagellar motor switch phosphatase FliY | COG1886 |
| 1148 | Flagellar biosynthesis protein FlhA | COG1298 |
| 1169 | Methyl-accepting chemotaxis sensory transducer | COG0840 |
| 1314 | Methyl-accepting chemotaxis protein | COG0840 |
| 1421 | Histidine kinase, HAMP region:Bacterial chemotaxis sensory transducer | COG0840 |
| 1462 | Methyl-accepting chemotaxis sensory transducer | COG0840 |
| 1488 | Methyl-accepting chemotaxis sensory transducer | COG0840 |
| 1489 | Putative uncharacterized protein | COG3143 |
| 1520 | Conserved domain protein | COG1749 |
| 1521 | Flagellar hook-associated protein FlgK | COG1256 |
| 1523 | Flagellar protein FlaF | COG5442 |
| 1524 | Putative uncharacterized protein | COG5443 |
| 1525 | Bacterial flagellin N-terminus domain protein | COG1344 |
| 1526 | Bacterial flagellin N-terminus domain protein | COG1344 |
| 1527 | Flagellin hook IN motif domain protein | COG1344 |
| 1528 | Bacterial flagellin N-terminus domain protein | COG1344 |
| 1531 | Putative uncharacterized protein | COG3951 |
| 1532 | Flagellar P-ring protein I | COG1706 |
| 1535 | Flagellar L-ring protein FlgH | COG2063 |
| 1536 | Flageller protein FlgA | COG1261 |
| 1537 | Flagellar basal-body rod FlgG | COG4786 |
| 1538 | Flagella basal body rod protein | COG4786 |
| 1539 | Flagellar basal body-associated protein FliL | COG1580 |
| 1540 | Flagellar motor switch protein FliM | COG1868 |
| 1544 | Flagellar transport protein FliP | COG1338 |
| 1546 | Flagellar basal-body rod protein B | COG1815 |
| 1547 | Flagellar basal-body rod protein C | COG1558 |
| 1548 | Flagellar hook-basal body protein FliE | COG1677 |
| 1549 | Flagellar biosynthesis protein Q | COG1987 |
| 1550 | Flagellar biosynthetic protein fliR | COG1684 |
| 1551 | Flagellar biosynthesis protein B | COG1377 |
| 1652 | Hpt domain protein | COG0643 |
| 1765 | Methyl-accepting chemotaxis protein signaling domain | COG0840 |
| 1798 | Putative surface presentation of antigens protein | COG1886 |
| 2432 | TPR repeat | COG3063 |
| 2758 | Methyl-accepting chemotaxis sensory transducer | COG0840 |
| 2761 | Methyl-accepting chemotaxis sensory transducer | COG0840 |
| 2762 | TPR repeat | COG3063 |
| 3052 | Methyl-accepting chemotaxis sensory transducer | COG0840 |
| 3438 | Chemotaxis protein | COG3143 |
| 3470 | Putative uncharacterized protein | COG3143 |
| 3606 | Type II secretory pathway, pseudopilin PulG | COG2165 |
| 3642 | Type IV pilus assembly protein PilZ | COG0840 |
| 3658 | Flp pilus assembly protein TadC | COG2064 |
| 3662 | Pilus biogenesis lipoprotein CpaD | COG5461 |
| 3709 | Methyl-accepting chemotaxis sensory transducer | COG0840 |
| 3710 | methyl-accepting chemotaxis sensory transducer (IMGterm | COG0840 |
| 3720 | Methyl-accepting chemotaxis sensory transducer | COG0840 |
| 3723 | methyl-accepting chemotaxis sensory transducer (IMGterm | COG0840 |
| 3742 | Methyl-accepting chemotaxis receptor/sensory transducer | COG0840 |
| 3849 | H^+^-transporting ATP synthase, flagellum-specific protein | COG1157 |
| 3851 | Flagellar basal body rod protein | COG4786 |
| 3853 | Chemotaxis transmembrane protein | COG1291 |
| 3854 | Flagellar motor switch protein FliM, putative | COG1868 |
| 3856 | Probable flagellar motor switch protein | COG1886 |
| 3857 | Flagellar motor switch protein FliG | COG1536 |
| 3858 | Flagellar biosynthetic protein FlhB | COG1377 |
| 3862 | Rod binding protein. | COG3951 |
| 3864 | Bacterial export protein, family 1 | COG1684 |
| 3865 | Flagellar biosynthesis transmembrane protein | COG1298 |
| 3869 | Flagellar biosynthesis transmembrane protein | COG1987 |
| 3870 | Flagellar hook capping protein | COG1843 |
| 3871 | Flagellum biosynthesis repressor | COG5443 |
| 3872 | Flagellar protein flaF | COG5442 |
| 3873 | Flagellar hook-associated protein L | COG1344 |
| 3874 | Flagellar hook-associated protein K | COG1256 |
| 3875 | Flagellar basal body FlaE | COG1749 |
| 3879 | Flagellar motor protein MotB | COG1360 |
| 3881 | Flagellar M-ring protein | COG1766 |
| 3882 | Flagellin-like protein | COG1344 |
| 3883 | Flagellar transport protein FliP | COG1338 |
| 3885 | Flagellar L-ring protein H | COG2063 |
| 3887 | Flagellar P-ring protein I | COG1706 |
| 3888 | Flagellar basal body P-ring biosynthesis protein-like protein | COG1261 |
| 3889 | Flagellar basal-body rod protein FlgG | COG4786 |
| 3890 | Flagellar hook-basal body complex protein FliE | COG1677 |
| 3891 | Flagellar basal-body rod protein FlgC | COG1558 |
| 3892 | Flagellar basal-body rod protein B | COG1815 |
| 3955 | Flagellin and hook associated protein | COG1344 |
| 3956 | Flagellar hook-associated protein | COG1256 |
| 3959 | FlgE protein | COG1749 |
| 3960 | Flagellar hook capping protein | COG1843 |
| 3963 | Flagellin-specific chaperone FliS | COG1516 |
| 3964 | Flagellar hook-associated 2-like | COG1345 |
| 3965 | Flagellin-like | COG1344 |
| p0042 | Conjugal transfer protein | COG5268 |
