# Supplementary material for: The Genome Sequence of Polymorphum gilvum SL003B-26A1T Reveals Its Genetic Basis for Crude Oil Degradation and Adaptation to the Saline Soil
Source: PLoS One. 2012 Feb 16;7(2):e31261. doi: 10.1371/journal.pone.0031261 (PMC3281065; doi:10.1371/journal.pone.0031261)
Supplement: Table S13 — Genes in cell motility and Chemotaxis (pathways via KO terms). (DOC) [file pone.0031261.s015.doc]

## Table S13 Genes in cell motility and Chemotaxis (pathways via KO terms)

| **Locus_Tag** | **Product Name** | **Func ID** | **Func Name** |
| --- | --- | --- | --- |
| 0016 | Methyl-accepting chemotaxis sensory transducer | KO:K03406 | methyl-accepting chemotaxis protein |
| 0489 | Flagellar hook-basal body protein | KO:K02390 | flagellar hook protein FlgE |
| 0490 | Flagellin-like protein | KO:K02406 | flagellin |
| 0841 | OmpA family protein | KO:K02557 | chemotaxis protein MotB |
| 0842 | MotA/TolQ/ExbB proton channel | KO:K02556 | chemotaxis protein MotA |
| 0898 | OmpA family protein | KO:K02557 | chemotaxis protein MotB |
| 0948 | CheA-like signal transduction histidine kinase | KO:K03407 | two-component system, chemotaxis family, sensor kinase CheA [EC:2.7.13.3] |
| 0949 | Probable purine-binding chemotaxis protein | KO:K03408 | purine-binding chemotaxis protein CheW |
| 0950 | Response regulator receiver domain protein (CheY-like) | KO:K03413 | two-component system, chemotaxis family, response regulator CheY |
| 0951 | Chemotaxis response regulator protein-glutamate methylesterase | KO:K03412 | two-component system, chemotaxis family, response regulator CheB [EC:3.1.1.61] |
| 0952 | CheR methyltransferase, SAM binding domain protein | KO:K00575 | chemotaxis protein methyltransferase CheR [EC:2.1.1.80] |
| 0955 | H^+^-transporting two-sector ATPase FliI | KO:K02412 | flagellum-specific ATP synthase [EC:3.6.3.14] |
| 0960 | ABC peptide transporter, periplasmic binding protein | KO:K12368 | dipeptide transport system substrate-binding protein |
| 1141 | Flagellar hook capping protein | KO:K02389 | flagellar basal-body rod modification protein FlgD |
| 1143 | Flagellar M-ring protein | KO:K02409 | flagellar M-ring protein FliF |
| 1144 | Flagellar motor switch protein FliG | KO:K02410 | flagellar motor switch protein FliG |
| 1145 | Flagellar assembly protein H | KO:K02411 | flagellar assembly protein FliH |
| 1146 | Flagellar motor switch phosphatase FliY | KO:K02417 | flagellar motor switch protein FliN/FliY |
| 1148 | Flagellar biosynthesis protein FlhA | KO:K02400 | flagellar biosynthesis protein FlhA |
| 1169 | Methyl-accepting chemotaxis sensory transducer | KO:K03406 | methyl-accepting chemotaxis protein |
| 1462 | Methyl-accepting chemotaxis sensory transducer | KO:K03406 | methyl-accepting chemotaxis protein |
| 1521 | Flagellar hook-associated protein FlgK | KO:K02396 | flagellar hook-associated protein 1 FlgK |
| 1532 | Flagellar P-ring protein I | KO:K02394 | flagellar P-ring protein precursor FlgI |
| 1535 | Flagellar L-ring protein FlgH | KO:K02393 | flagellar L-ring protein precursor FlgH |
| 1536 | Flageller protein FlgA | KO:K02386 | flagella basal body P-ring formation protein FlgA |
| 1537 | Flagellar basal-body rod FlgG | KO:K02392 | flagellar basal-body rod protein FlgG |
| 1538 | Flagella basal body rod protein | KO:K02391 | flagellar basal-body rod protein FlgF |
| 1540 | Flagellar motor switch protein FliM | KO:K02416 | flagellar motor switch protein FliM |
| 1544 | Flagellar transport protein FliP | KO:K02419 | flagellar biosynthetic protein FliP |
| 1546 | Flagellar basal-body rod protein B | KO:K02387 | flagellar basal-body rod protein FlgB |
| 1547 | Flagellar basal-body rod protein C | KO:K02388 | flagellar basal-body rod protein FlgC |
| 1548 | Flagellar hook-basal body protein FliE | KO:K02408 | flagellar hook-basal body complex protein FliE |
| 1549 | Flagellar biosynthesis protein Q | KO:K02420 | flagellar biosynthetic protein FliQ |
| 1550 | Flagellar biosynthetic protein fliR | KO:K02421 | flagellar biosynthetic protein FliR |
| 1551 | Flagellar biosynthesis protein B | KO:K02401 | flagellar biosynthetic protein FlhB |
| 1798 | Putative surface presentation of antigens protein | KO:K02417 | flagellar motor switch protein FliN/FliY |
| 2758 | Methyl-accepting chemotaxis sensory transducer | KO:K03406 | methyl-accepting chemotaxis protein |
| 3439 | Response regulator receiver (CheY-like protein) | KO:K03413 | two-component system, chemotaxis family, response regulator CheY |
| 3710 | Histidine kinase, HAMP region:Bacterial chemotaxis sensory transducer | KO:K03406 | methyl-accepting chemotaxis protein |
| 3742 | Methyl-accepting chemotaxis receptor/sensory transducer | KO:K03406 | methyl-accepting chemotaxis protein |
| 3849 | H(+)-transporting ATP synthase, flagellum-specific protein | KO:K02412 | flagellum-specific ATP synthase [EC:3.6.3.14] |
| 3851 | Flagellar basal body rod protein | KO:K02391 | flagellar basal-body rod protein FlgF |
| 3853 | Chemotaxis transmembrane protein | KO:K02556 | chemotaxis protein MotA |
| 3854 | Flagellar motor switch protein FliM, putative | KO:K02416 | flagellar motor switch protein FliM |
| 3857 | Flagellar motor switch protein FliG | KO:K02410 | flagellar motor switch protein FliG |
| 3858 | Flagellar biosynthetic protein FlhB | KO:K02401 | flagellar biosynthetic protein FlhB |
| 3864 | Bacterial export protein, family 1 | KO:K02421 | flagellar biosynthetic protein FliR |
| 3865 | Flagellar biosynthesis transmembrane protein | KO:K02400 | flagellar biosynthesis protein FlhA |
| 3869 | Flagellar biosynthesis transmembrane protein | KO:K02420 | flagellar biosynthetic protein FliQ |
| 3870 | Flagellar hook capping protein | KO:K02389 | flagellar basal-body rod modification protein FlgD |
| 3873 | Flagellar hook-associated protein L | KO:K02397 | flagellar hook-associated protein 3 FlgL |
| 3874 | Flagellar hook-associated protein K | KO:K02396 | flagellar hook-associated protein 1 FlgK |
| 3875 | Flagellar basal body FlaE | KO:K02390 | flagellar hook protein FlgE |
| 3879 | Flagellar motor protein MotB | KO:K02557 | chemotaxis protein MotB |
| 3881 | Flagellar M-ring protein | KO:K02409 | flagellar M-ring protein FliF |
| 3882 | Flagellin-like protein | KO:K02406 | flagellin |
| 3883 | Flagellar transport protein FliP | KO:K02419 | flagellar biosynthetic protein FliP |
| 3885 | Flagellar L-ring protein H | KO:K02393 | flagellar L-ring protein precursor FlgH |
| 3887 | Flagellar P-ring protein I | KO:K02394 | flagellar P-ring protein precursor FlgI |
| 3888 | Flagellar basal body P-ring biosynthesis protein-like protein | KO:K02386 | flagella basal body P-ring formation protein FlgA |
| 3889 | Flagellar basal-body rod protein FlgG | KO:K02392 | flagellar basal-body rod protein FlgG |
| 3890 | Flagellar hook-basal body complex protein FliE | KO:K02408 | flagellar hook-basal body complex protein FliE |
| 3891 | Flagellar basal-body rod protein FlgC | KO:K02388 | flagellar basal-body rod protein FlgC |
| 3892 | Flagellar basal-body rod protein B | KO:K02387 | flagellar basal-body rod protein FlgB |
| 3955 | Flagellin and hook associated protein | KO:K02397 | flagellar hook-associated protein 3 FlgL |
| 3956 | Flagellar hook-associated protein | KO:K02396 | flagellar hook-associated protein 1 FlgK |
| 3959 | FlgE protein | KO:K02390 | flagellar hook protein FlgE |
| 3960 | Flagellar hook capping protein | KO:K02389 | flagellar basal-body rod modification protein FlgD |
| 3964 | Flagellar hook-associated 2-like | KO:K02407 | flagellar hook-associated protein 2 |
| 3965 | Flagellin-like | KO:K02406 | flagellin |
